# The basic helix–loop–helix (bHLH) transcription factor *DTT1* is part of a paired key that unlocks the tapetum transition in barley anther development

**DOI:** 10.1093/plcell/koaf230

**Published:** 2025-09-30

**Authors:** Miaoyuan Hua, Wenzhe Yin, Alison C Tidy, José Fernández Gómez, Huanjun Li, Shuya Shi, Guangwei Xing, Jie Zong, Zoe A Wilson

**Affiliations:** Division of Plant and Crop Sciences, School of Biosciences, University of Nottingham, Sutton Bonington Campus, Loughborough, Leicestershire LE12 5RD, UK; School of Agriculture and Biology, Shanghai Jiao Tong University, Shanghai 200240, China; School of Life Sciences and Biotechnology, Shanghai Jiao Tong University, Shanghai 200240, China; Hainan Research Institute, Shanghai Jiao Tong University, Sanya 572024, China; Division of Plant and Crop Sciences, School of Biosciences, University of Nottingham, Sutton Bonington Campus, Loughborough, Leicestershire LE12 5RD, UK; Division of Plant and Crop Sciences, School of Biosciences, University of Nottingham, Sutton Bonington Campus, Loughborough, Leicestershire LE12 5RD, UK; Research and Development, Empyrean Neuroscience, Cambridge CB22 3AT, UK; School of Life Sciences and Biotechnology, Shanghai Jiao Tong University, Shanghai 200240, China; Division of Plant and Crop Sciences, School of Biosciences, University of Nottingham, Sutton Bonington Campus, Loughborough, Leicestershire LE12 5RD, UK; Faculty of Synthetic Biology, Shenzhen Institute of Advanced Technology, Shenzhen, Guangdong 518055, P.R. China; Key Laboratory of Quantitative Synthetic Biology, Shenzhen Institute of Synthetic Biology, Shenzhen Institute of Advanced Technology, Chinese Academy of Sciences, Shenzhen, Guangdong 518055, P.R. China; NovelBio Bio-Pharm Technology CO., Ltd, Shanghai 201112, China; Division of Plant and Crop Sciences, School of Biosciences, University of Nottingham, Sutton Bonington Campus, Loughborough, Leicestershire LE12 5RD, UK

## Abstract

The production of viable pollen is essential for effective fertilization and optimal crop yields; however, our understanding of the underlying mechanisms remains limited. Here, we characterize a barley (*Hordeum vulgare*) anther basic helix–loop–helix (bHLH) gene, *DEFECTIVE TAPETUM TRANSITION1* (*DTT1*), a gatekeeper that regulates tapetum development. The *dtt1* mutant is male sterile, failing to acquire tapetum cell fate identity with overproliferation of indeterminate tapetal precursor cells, a lack of tapetum endomitosis, and cell wall degeneration. DTT1 forms heterodimers with DYSFUNCTIONAL TAPETUM1 (HvDYT1) through bHLH and ACT-like(BIF) domains, with the ACT-like(BIF) domain and the IKL motif being critical in partner selection. These heterodimers may subsequently interact with each other through the bHLH-ACT-like(BIF) domain to activate expression. Transcriptome analysis confirmed that anther development transition from stages 6 to 7 fails in *dtt1*. We show that HvTDF1-related pathways are downstream of DTT1 and work in independent and overlapping networks with other conserved tapetum regulators. SELEX-seq analysis indicates that DTT1 can bind to DNA with a chimeric or canonical E-box motif only when it forms a complex with HvDYT1. In vivo dual-luciferase assays confirmed that the DTT1–HvDYT1 complex directly regulates the expression of several stage 7-specific transcription factors, such as *HvTDF1*, *HvEAT1*, and the identified *GAMYB* target genes. Therefore, the paired DTT1–HvDYT1 complex appears crucial in orchestrating the transition of tapetum cell fate by modulating genes involved in diverse biological pathways. This work uncovers detailed relationships in barley tapetum regulation and male fertility.

## Teaser

DTT1 acts as a gatekeeper regulating tapetum cell fate identity, development, and pollen formation by forming heterodimers with HvDYT1 and modulating genes in diverse pollen development pathways.

## Introduction

In flowering plants, the anther is the male organ that supports the development and release of pollen grains, which are essential for successful pollination and seed formation. Barley (*Hordeum vulgare*) is one of the most widespread crops grown globally; however, despite the potential for yield enhancement through hybrid breeding and the associated need to control male fertility in this autogamous cereal, understanding of the process of male reproduction is still in its infancy. Anther development in model species has been classified into 14 distinct stages based on specific cell morphologies (Sanders et al. 1999; Zhang et al. 2011). Anther primordia have 3 distinct cell layers, which are L1, L2, and L3 from outermost to innermost, which differentiate into specific cell layers required for pollen formation. At stage 2, L2 cells develop into the archesporial cells, which start the further transition via periclinal and asymmetrical divisions, to produce sporogenesis cells and primary parietal cells (PPCs) at stage 3. Sporogenesis cells start the germline lineage to generate mature pollen grains at the following stages. The PPCs divide into inner and outer secondary parietal cells (SPCs) at stage 4. The outer SPC then generates the endothecium, and the inner one divides into the tapetum and middle layer, respectively, at stage 5.

Several plasma membrane-localized leucine-rich receptor-like kinases, such as *EXCESS MICROSPOROCYTES1* (*EMS1*), also called *EXTRA SPOROGENOUS CELLS*, and its coreceptor, *SOMATIC EMBRYOGENESIS RECEPTOR-LIKE KINASE1* (*SERK1*) and *SERK2*, have been shown to control the division of the SPC into the tapetum layer (Yang et al. 2003; Albrecht et al. 2005; Colcombet et al. 2005; Chang et al. 2011). *TAPETUM DETERMINANT1* (*TPD1*) encodes for a small peptide that is principally expressed in microsporocytes and thought to be secreted into the interface between male reproductive cells and tapetal cells (Yang et al. 2003). TPD1 was identified as a ligand that interacts with the EMS1–SERK receptor complex and determines tapetal layer formation (Jia et al. 2008; Grelon et al. 2016). Mutants of these genes show similar phenotypes with a lack of tapetum and extra microsporocytes. The molecular function of the middle layer, the other cell layer that also originates from the SPC, has rarely been studied. *RECEPTOR-LIKE KINASE2* (*RPK2*) encodes a receptor-like kinase that is required for middle layer formation, *rpk2* mutants lack the middle layer and are male sterile due to failed microspore development after release from tetrads (Mizuno et al. 2007). CLAVATA3 INSENSITIVE RECEPTOR KINASEs (CIKs) have been shown to be a coreceptor protein that works together with RPK2 to determine middle layer formation in *Arabidopsis* (Cui et al. 2018). Along with the mechanism that determines the pattern of anther somatic cell layers, the cell number of each cell type is also tightly regulated. Studies conducted in *Arabidopsis* showed that outer somatic layers, epidermis, and endothecium, stopped cell division at stage 10 (Xue et al. 2021). Two inner somatic cell layers, the middle layer and tapetum, stop cell division at stages 6 and 7, respectively, and then undergo further development processes during the following stages (Xue et al. 2021; Wu et al. 2023). This phenomenon is consistent with their biological roles in anther development; both layers subsequently degenerate.

The biological function of the tapetum is tightly regulated through a conserved regulatory genetic pathway, DYSFUNCTIONAL TAPETUM1 (DYT1)–DEFECTIVE IN TAPETAL DEVELOPMENT AND FUNCTION 1 (TDF1)-ABORTED MICROSPORES (AMS)–MYB80–MALE STERILITY 1 (MS1), which has been characterized in some monocot and dicot plants (Higginson et al. 2003; Sorensen et al. 2003; Jung et al. 2005; Zhang et al. 2006, 2007; Ito et al. 2007; Yang et al. 2007; Zhu et al. 2008; Xu et al. 2010; Li et al. 2011; Phan et al. 2011; Moon et al. 2013; Fernández Gómez and Wilson 2014; Cai et al. 2015; Hua et al. 2023). Interestingly, if the genes are classified based on their gene families, this genetic pathway consists of a repeated bHLH-MYB cassette. Both *DYT1* and *AMS* genes encode for bHLH transcription factors (TFs). *TDF1* and *MYB80* are MYB TFs. Previous gene family analysis of bHLH genes in *Arabidopsis* showed a close relationship between DYT1 and AMS (Heim et al., 2003; Toledo-Ortiz et al. 2003). TDF1 and MYB80 also group into the same subclade in the MYB family phylogenetic analysis (Dubos et al. 2010). These results suggest that a potential gene duplication event happened prior to these genes gaining divergent functions.

Additionally, 3 functionally redundant bHLH proteins, bHLH010, bHLH089, and bHLH091, have been identified in *Arabidopsis*, which interact with DYT1 and AMS to form heterodimers, to promote specific downstream gene expression, and to play important roles in adapting to dynamic environment conditions (Xu et al. 2010; Zhu et al. 2015; Cui et al. 2016; Lai et al. 2022, 2023; Robson et al. 2023). However, in some monocot plants, only 2 bHLH proteins are identified as homologous to *Arabidopsis* bHLH10/091, such as TIP2 and EAT1 (DTD) in rice (Ji et al. 2013; Niu et al. 2013; Fu et al. 2014; Ko et al. 2014). Their orthologous genes, *MS23* and *ZmbHLH122*, were also reported in maize (Nan et al. 2016). Studies conducted in maize and rice indicate similar functions, with these bHLH proteins also forming heterodimers with orthologs of DYT1 and AMS (Nan et al. 2016; Ono et al. 2018), with the HLH region mediating homodimer and heterodimer formation. The basic region, an α helix, is in front of the helix–loop–helix (HLH) and is responsible for recognizing the canonical E-box (^5′^CANNTG^3′^) DNA motif. These complexes regulate tapetum 24-nt phasiRNA biogenesis by activating endoribonuclease (Dcl5) expression to cleave 24-nt precursors (Ono et al. 2018; Nan et al. 2022). The tapetum-originated 24-nt phasiRNA then plays key roles in pollen development (Ono et al. 2018; Nan et al. 2022). Studies in rice also showed a direct relationship between these bHLH proteins and tapetum programmed cell death (PCD) and pollen wall formation (Ji et al. 2013; Niu et al. 2013; Fu et al. 2014).

We previously reported HvTDF1 as a transcription activator that determines barley tapetum development by regulating osmotin protein expression and discussed the relationship between the barley tapetum development pathway and the TFs HvTDF1/HvAMS (Hua et al. 2023). To further clarify tapetum development in barley, we focused on analysis of orthologs of tapetum-specific bHLH TFs. Here, we identify a barley anther bHLH protein, DEFECTIVE TAPETUM TRANSITION1 (DTT1), as a gatekeeper regulating tapetum development through linearized and parallel pathways. The *dtt1* knockout line showed failed tapetum cell fate acquisition with overproliferation of indeterminate tapetal cells. The transcriptome data of *dtt1* mutant also suggest that anther development failed the transition from stage 6 to stage 7. Transcriptome comparison between the *dtt1* mutant and *Hvtdf1* mutant indicates that HvTDF1-regulated pathways are downstream of DTT1 and working together with other conserved regulators in a parallel and overlapping manner. DTT1 protein tends to form a complex with HvDYT1 rather than homodimerization. DTT1–HvDYT1 complex activates gene expression through a dual-E-box motif. This work uncovers detailed relationships in the tapetum regulatory genetic pathway in barley.

## Results

### Diversity between barley tapetum bHLH proteins and other species

Numerous bHLH TFs have been characterized in anther tapetum development in monocot and dicot plants (Sorensen et al. 2003; Jung et al. 2005; Li et al. 2006; Zhang et al. 2006; Ji et al. 2013; Moon et al. 2013; Niu et al. 2013; Fu et al. 2014; Ko et al. 2014; Zhu et al. 2015; Nan et al. 2016; Ono et al. 2018; Zheng et al. 2020). These include both orthologous and divergent genes in dicot and monocots, for example, the orthologous *DYT1* and *AMS* genes reported in *Arabidopsis*, rice, and maize (Sorensen et al. 2003; Jung et al. 2005; Li et al. 2006; Zhang et al. 2006; Nan et al. 2016). Three functionally redundant bHLH proteins, bHLH010, bHLH089, and bHLH091, were reported in *Arabidopsis* (Zhu et al. 2015); however, only 2 paralog bHLH proteins (TIP2 and EAT1) were found to play important roles in tapetum cell fate determination and PCD, respectively, in some cereal crops, including rice and maize (Ji et al. 2013; Niu et al. 2013; Fu et al. 2014; Nan et al. 2016).

**Figure 1.**
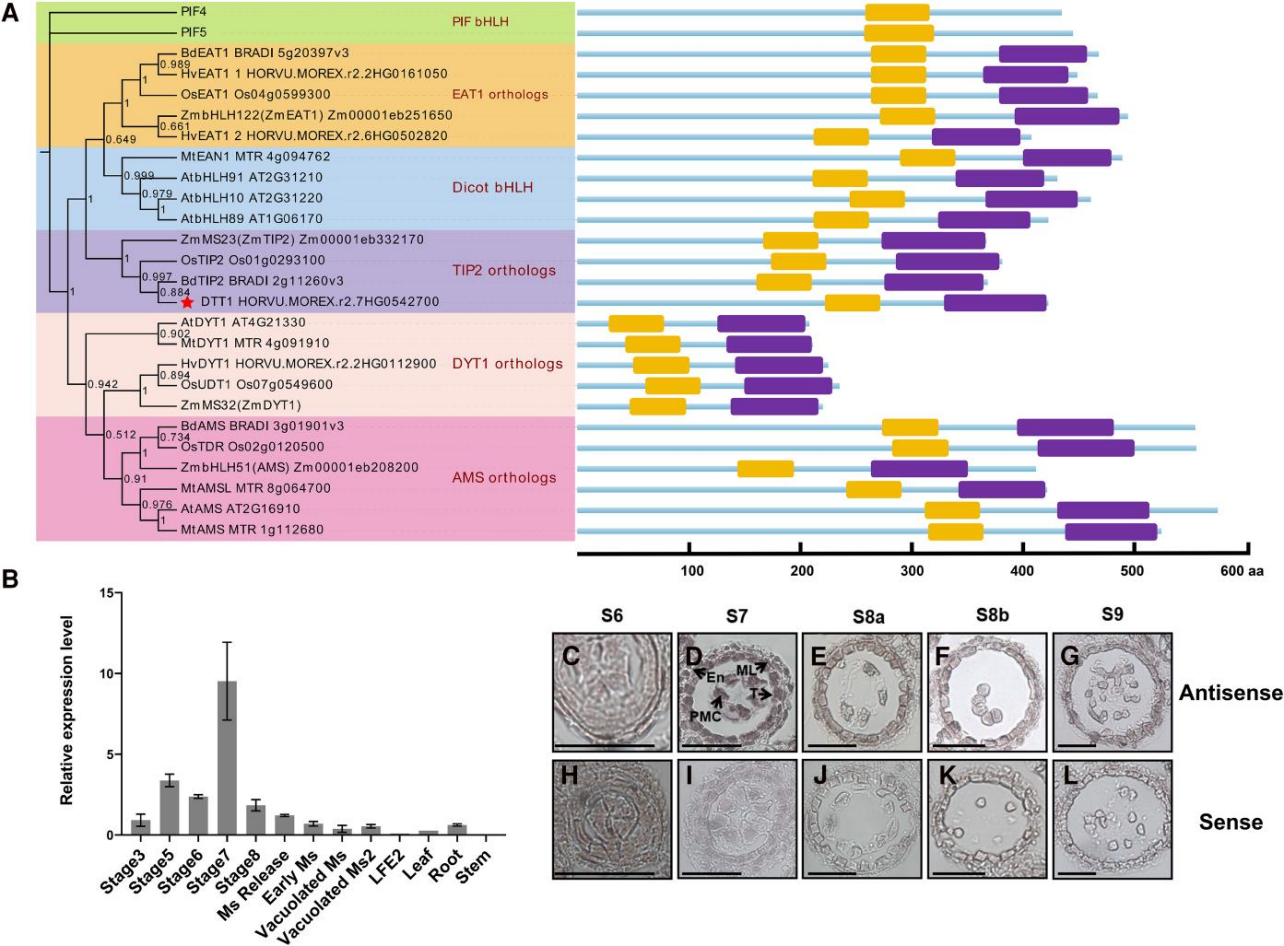
Phylogenetic and expression analyses of tapetum-specific bHLH genes during the anther development in barley. **A)** Phylogenetic analysis of bHLH genes, clusters shown with different colors. On the right, protein secondary structure is shown, highlighted by colors indicating the different protein secondary structures. Yellow = bHLH domain; purple = ACT-like(BIF) domain. The numbers on phylogenetic analysis indicate the bootstrap value. **B)** RT-qPCR analysis of *DTT1* expression from different tissues and stages of development, RNA was extracted from the whole spike and other organs. Staging based on Fernández Gómez and Wilson (2012); Ms: microspore; LFE2: last flag sheath extended 5 to 10 cm. Error bars refer to SD of 2 biological replicates. **C–G)** RNA in situ hybridization by *DTT1*-specific antisense probe. T, tapetum; ML, middle layer; En, endothecium; PMC, pollen mother cell. Bars = 50 µm. **H–L)** RNA in situ hybridization by *DTT1*-specific sense probe. T, tapetum. Bars = 50 µm. **C** and **H)** Primary sporogenous cells. Three cell layers surrounding the anther locule. **D** and **I)** Secondary sporogenous cells to PMCs. Four layers surrounding the anther locule: epidermis, endothecium (En), middle layer (ML), and tapetum (T). **E** and **J)** PMCs undergo meiosis. Tapetum layer is prominent. **F** and **K)** Microspores released from the tetrad. Tapetum is vacuolated. **G** and **L)** Free microspore stage. Middle layer undergoes crushing. The prominent tapetum layer starts to degenerate.

Our previous study on the barley *TDF1* orthologous gene *HvTDF1* indicated that tapetum development had both conserved and unique aspects between monocot and dicots (Hua et al. 2023). We therefore also looked at the orthologs of the bHLH genes in barley to determine the level of diversity. Barley DYT1 and AMS showed specific expression patterns corresponding to tapetum development (Hua et al. 2023). We also tried to identify additional bHLH genes, such as putative barley TIP2 and EAT1. Interestingly, barley showed different numbers of these 2 genes compared with other typical monocots. Phylogenetic analysis indicates that 2 proteins are putative EAT1 orthologs and 1 for TIP2 (Fig. 1A). We predicted the protein secondary structure for each bHLH TF, and all have ACT-like(BIF) domain, ββαββα topology, at their C-terminal regions, but in *Arabidopsis* PIF4 and PIF5 bHLH proteins, no such topology was identified (Fig. 1A). Functional studies on the ACT-like(BIF) domain have demonstrated that it plays a critical role in homodimer or heterodimer formation separate from the bHLH domain (Feller et al. 2006; Kong et al. 2012; Cui et al. 2016; Lee et al. 2021). The DYT1 ACT-like(BIF) domain is also important for subcellular localization and heterodimer formation with other bHLH proteins. The resultant complexes can strongly activate downstream gene expression to ensure normal anther development (Cui et al. 2016).

### Determining putative bHLH ortholog gene expression pattern in barley

To study the putative function of these barley bHLH genes, we identified their expression patterns from our barley staged anther RNA-seq data (Hua et al. 2023). Both barley *EAT1* orthologs, *HvEAT1-L1* and *HvEAT1-L2*, share similar expression patterns as reported in rice with 2 peaks, while the *TIP2* ortholog (*DTT1*) showed broad expression from anther stage 6 to stage 8b (Supplementary Fig. S1, A to C). We confirmed the *TIP2* ortholog (*DTT1*) expression pattern by detailed RT-qPCR using barley spike samples covering early to late anther development stages (Fig. 1B). This indicated that the barley *TIP2* ortholog (*DTT1*) has prolonged expression from stage 3 to single-microspore release, with a peak at stage 7 (Fig. 1B). To further determine its temporal and spatial expression patterns within the anther, we conducted RNA in situ hybridization (Fig. 1, C to L). Signals could be detected from the tapetum, middle layer, endothecium, and epidermis (Fig. 1D). These results suggest that the barley putative *TIP2* ortholog (*DTT1*) may be involved in multiple biological processes across anther development.

### CRISPR-CAS9-generated barley *dtt1* mutant showed male sterility

**Figure 2.**
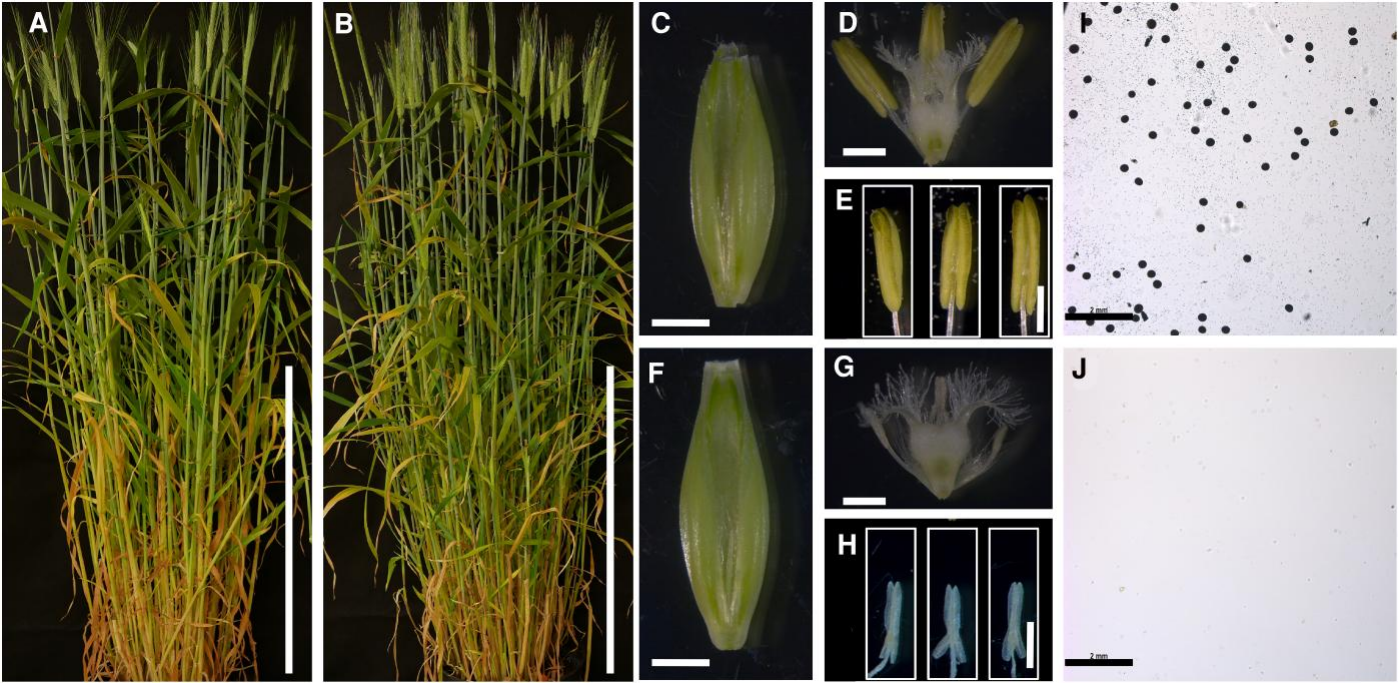
Comparison between wild-type barley and the *dtt1-1* mutant in the cv. Golden Promise. **A** and **B)** Whole-plant morphology, **A)** wild type, **B)**  *dtt1-1*. Bar = 50 cm. **C–H)** Comparative morphology of floral tissues of wild type **C–E)** and *dtt1-1*  **F–H)**, showing florets **C, F)**, with lemma and palea removed **D, G)**, and anthers **E, H)**. Bar = 1 mm. **I–J)** KI-I2 pollen viability staining, **I)** wild type, **J)**  *dtt1-1*. Bar = 2 mm.

Barley mutants were generated by a barley-specific CRISPR-CAS9 system (Lawrenson et al. 2015); 3 different targets were selected and assembled into 1 expression vector (Supplementary Fig. S2A). In total, 39 independent transgenic plants were obtained, and 2 alleles were confirmed by Sanger sequencing. The first allele had a 229-bp deletion between target 2 and target 3 (Supplementary Fig. S2, B and C). The other allele had a 2-bp deletion at target 2 and a 1-bp insertion at target 3 (Supplementary Fig. S2D). Both alleles were backcrossed with the wild-type Golden Promise to minimize potential off-target mutations and subsequently maintained by self-pollination. Both alleles showed similar phenotypes, appearing as wild type in their vegetative state but male sterile in homozygous mutants (Fig. 2; Supplementary Fig. S3). The spike lengths of both mutants were consistent with wild type (Supplementary Fig. S3G); thus, our spike length-based anther staging system (Fernández Gómez and Wilson 2012) was used to identify specific developmental stages. The 229-bp deletion mutant was selected for further detailed study.

**Figure 3.**
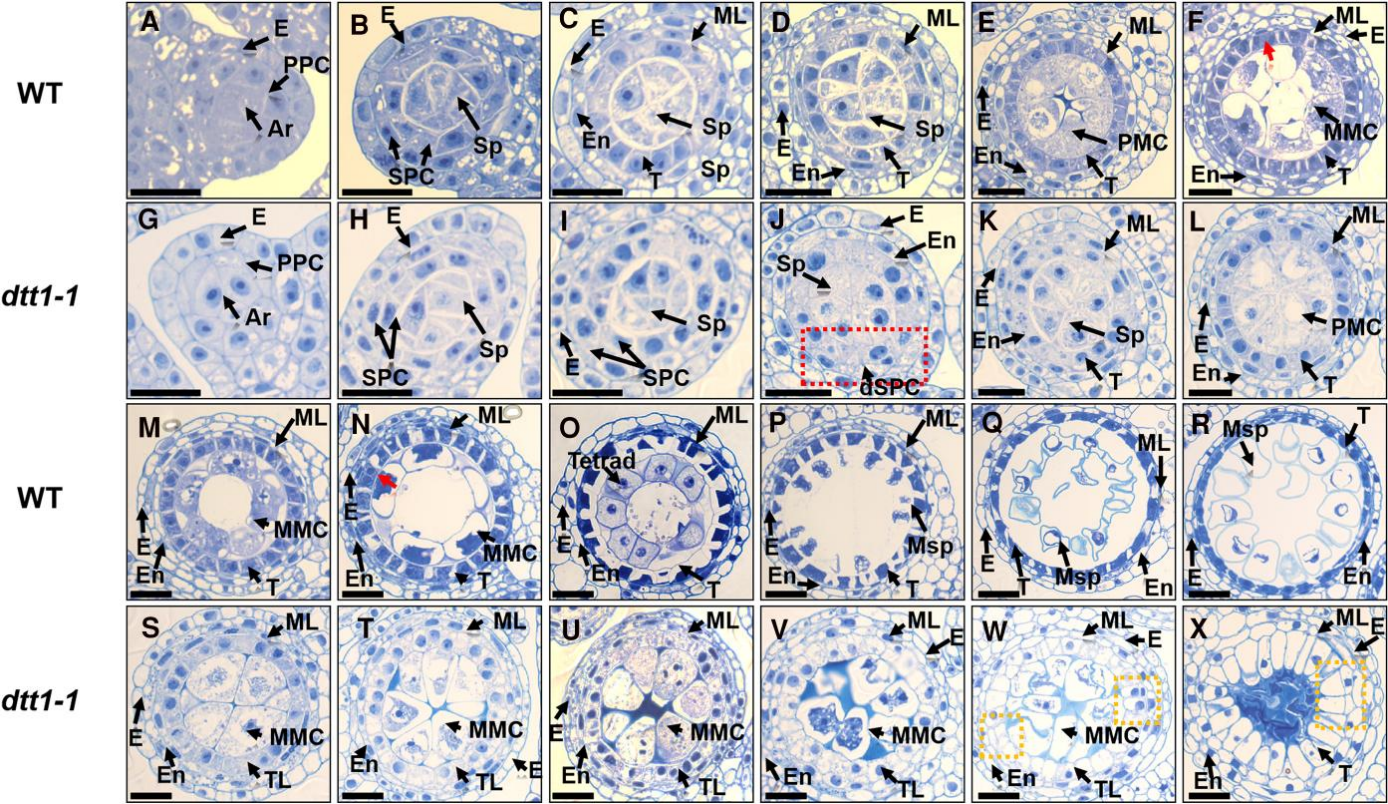
Transverse semi-thin sections showing the comparison between wild type and the *dtt1-1* mutant. Barley spikes collected from stage 3 to stage 10 from wild-type and the *dtt1-1* mutant lines. A–F and M–R are single wild-type anther locules, the rest **(G** to **L** and **S** to **X)** are equivalent developmental stages of *dtt1-1*. **A** and **G)** Stage 3; **B** and **H)** stage 4; **C** and **I)** stage 5; **D** and **J)** stage 6; **E** and **K)** stage 7; **F, M, N** and **L, S, T)** stage 8a; **O** and **U)** stage 8b; **P–R** and **V–X)** stage 9 and stage 10. Red dotted line box (J) indicates the dividing SPCs of *dtt1* mutant. Orange dotted line boxes **(W, X)** indicate the irregular divisional innermost cell layer of *dtt1* mutant. Red arrow **(F, N)** indicates the endomitosis in the tapetum cells. E, epidermis; En, endothecium; ML, middle layer; T, tapetum; Msp, microspore; MP, mature pollen; PPC, primary parietal cell; SPC, secondary parietal cell; dSPC, dividing SPC; Sp, sporogenous cell; PMC, pollen mother cell; MMC, meiotic microspore cell; TL, tapetum-like. Bars = 25 μm.

To characterize the anther locule defects, we conducted transverse semi-thin sectioning of the *dtt1-1* mutant and wild type. Early anther development progressed normally; archesporial cells underwent periclinal divisions to generate the somatic PPC layer at stage 3. At stage 4, the PPC layer generated the 2 SPC cell layers, in which the inner one divided into tapetum and middle layer and the outer differentiated as endothecium layer at stage 5. At this point, the 4 anther somatic cell layers that surround the sporogenesis cells were established. In the initiation of stage 6, all anther cell types form more daughter cells through cell division. Stage 7 is the initiation stage of tapetal cell fate acquisition and pollen mother cell (PMC) meiosis. In wild type, these events sequentially occurred (Fig. 3, A to E). The *dtt1-1* mutant was able to successfully complete cell layer establishment (Fig. 3, G to K); however, afterwards, it showed similar morphology as wild-type stage 6, but with faint staining in the tapetum layer and no obvious callose accumulated in the PMC region (Fig. 3L). During the following stages, wild-type plants underwent meiosis and formed tetrads at stage 8b (Fig. 3, F and M–O); the tapetum acquired its cell fate during this process, with obvious endomitosis occurring in the tapetum cells (Fig. 3N). After single-microspore release from the tetrad at stage 9, the tapetum layer started PCD, which provides the essential nutrients and materials for the developing microspores, and became thinner with darker-stained cytoplasm (Fig. 3, P to R). During these stages, the mutant anthers showed irregular somatic cell layer development, in which the tapetum did not appear to acquire the normal cell fate, endomitosis was not observed at any of the following developmental stages, and the staining pattern remained as in wild-type stage 6 (Fig. 3, S to V). The tapetum-like layer in the mutant retained cell division ability, with additional cell divisions occurring prior to microspore degeneration (Fig. 3, W and X). PMC nuclei started the meiosis process with chromosome condensation occurring in the mutant meiotic microspore cells (MMCs) but did not show reorganization of the nuclei back to a round shape in the subsequent development stages (Fig. 3, S to W); MMCs were subsequently engulfed by the irregularly developed tapetum. Based on the phenotype of irregular tapetum transition from stages 6 to 7, we termed this *bHLH* gene as *DTT1*.

**Figure 4.**
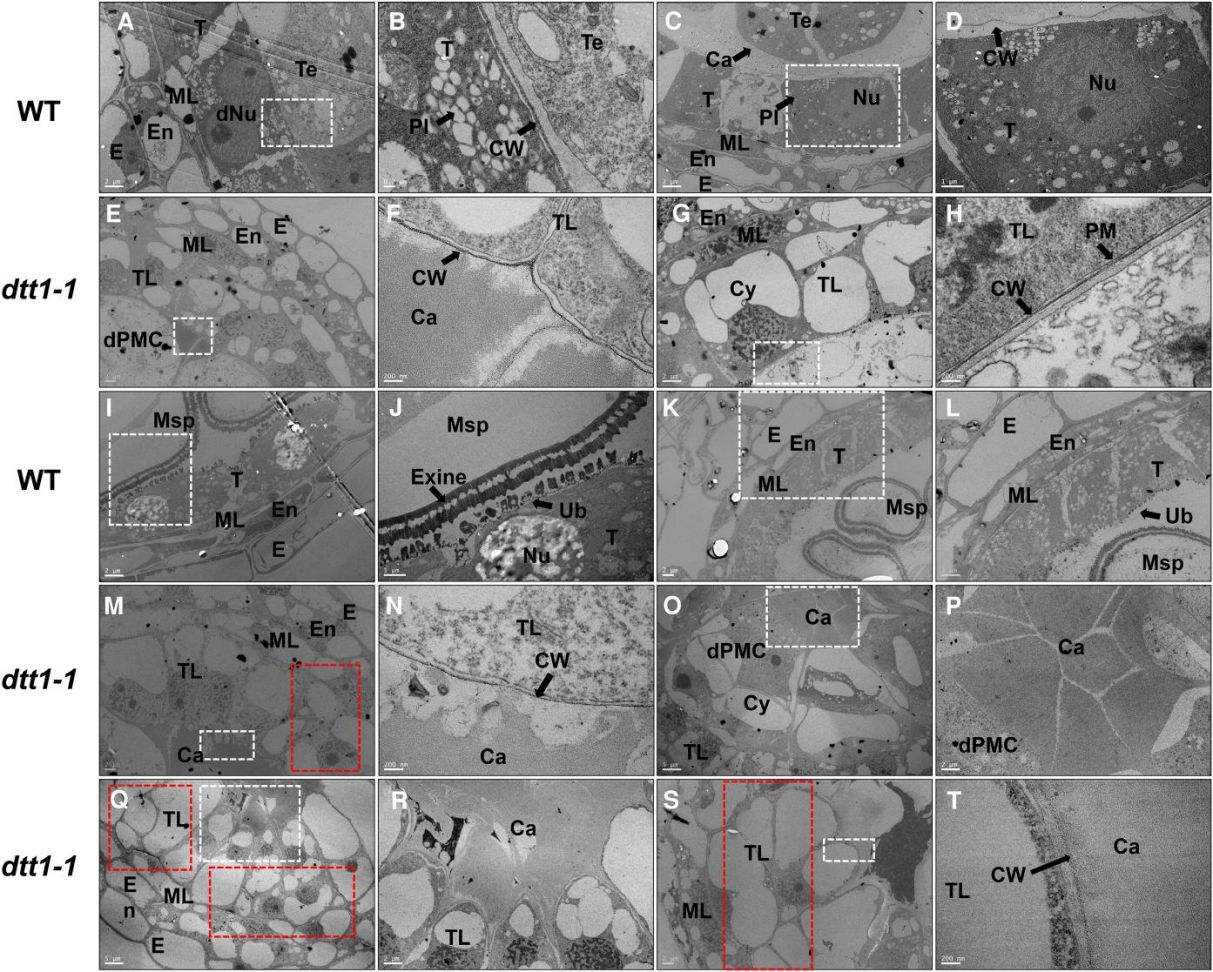
TEM analysis of wild type and the *dtt1* mutant. The wild type **A** and **C)** and *dtt1-1* mutant **E** and **G)** refer to anther transverse sections at stage 8b. **B** and **D)** Showing the higher magnification of the white box region in **A** and **C**; **F** and **H)** showing the higher magnification of the white box of **E** and **G)**, respectively. **I** and **K)** Representing the early single-microspore stage and later single-microspore stage of wild type, respectively. **J** and **L)** Showing the higher magnification of **I** and **K**. **M** and **O)** Referring to the tapetum layer and central region of the early single-microspore stage of the *dtt1-1* mutant. **N** and **P)** Showing the higher magnification of **M** and **O**. **Q** and **S)** Representing the cell division in the tapetum layer at the later single-microspore stage of the *dtt1-1* mutant. **R** and **T)** The higher magnifications of Q and S, respectively. Red boxes are showing the cell division regions in the tapetum layer of *dtt1*. E, epidermis; En, endothecium; ML, middle layer; T, tapetum; dNu, double nuclei; Nu, nuclei; PI, plastid; Te, tetrad; CW, cell wall; Ca, callose; dPMC, defective PMC; Cy, cytoplasm; TL, tapetum-like; PM, plasma membrane; Msp, microspore; Ub, Ubisch body. Bars = 2 μm in A, C, E, G, I, K, M, O–R, and S. Bars = 200 nm in B, F, H, L, N, and T. Bars = 5 μm in **O** and **Q**. Bars = 1 μm in **J** and **D**.

We examined the *dtt1-1* mutant by transmission electron microscopy (TEM) to investigate the tapetum transition defect. Samples were collected from tetrad, early single-microspore, and late single-microspore stages from wild type and the mutant. In the wild-type tetrad stage, the tapetum cell layer completed endomitosis with 2 nuclei visible in 1 tapetum cell (Fig. 4A). The tapetum cell wall remained, and tiny round-shaped deposits and organelles were seen in the tapetum cells (Fig. 4, B to D). In the *dtt1* mutant, no tapetum endomitosis was observed, and the tapetum cells were occupied by an enlarged vacuole without other obvious organelles (Fig. 4, E, F, G, and H). In the wild type at early and late single-microspore stages, the microspore exine formed, and Ubisch bodies were visible on the inner tapetum surface, instead of the cell wall structure (Fig. 4, I to L). Interestingly, the cell wall remained in the *dtt1* mutant, and callose accumulated in the middle of the anther locule (Fig. 4, M to P). The tapetum-like layer in *dtt1* showed additional cell divisions visible in both early and late single-microspore stages (Fig. 4, Q to T), but no endomitosis. Aniline blue staining indicated that increased callose accumulated in the anther locule and surrounded the irregularly developed MMCs in *dtt1* at stages 8b and 9 (Supplementary Fig. S4).

### DTT1 preferentially forms heterodimers with other bHLH TFs

**Figure 5.**
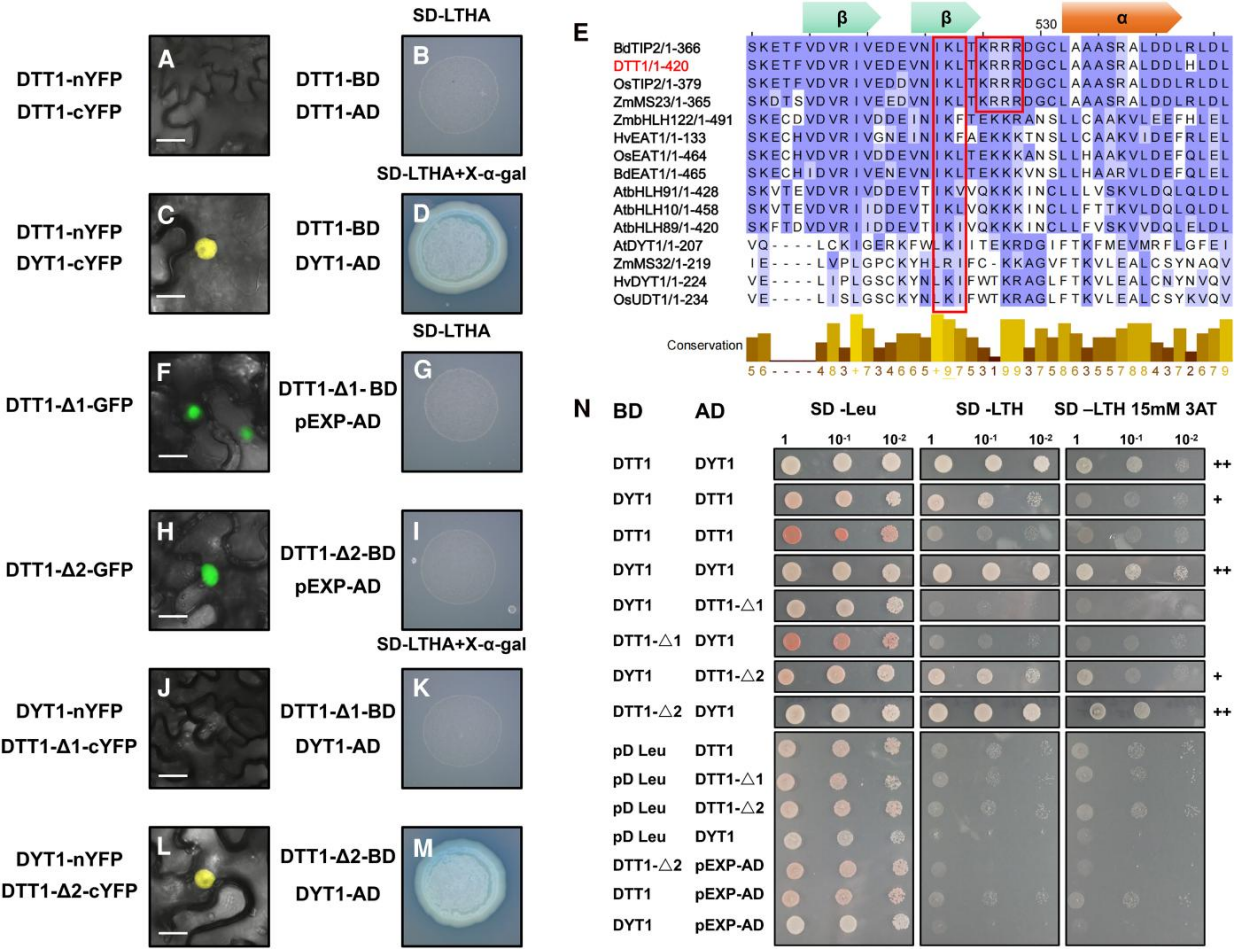
DTT1 preferentially forms a complex with HvDYT1 rather than self-dimerization. **A** and **C)** Full-length native protein BiFC assay results in *N. benthamiana* leaves. Bars = 20 μm. **A)** Showing lack of self-interaction of DTT1. **C)** YFP signal indicating the interaction between DTT1-nYFP and HvDYT1-cYFP. **B** and **D)** Full-length native proteins in Y2H assay. **B)** Showing lack of self-interaction of DTT1. **D)** Showing interaction between DTT1 and HvDYT1. **E)** Protein sequence alignment from reported key bHLH proteins in monocot and dicot species. Boxes indicate the 2 highly conserved regions IKL and KRRR, green arrows indicate β strand, and the orange arrow indicates α helix. **F** and **H)** Subcellular localizations of **F)** DTT1-delta1(△1) and **H)** DTT1-delta2(△2), proteins which were mutated in their conserved regions IKL to DQD and KRRR to CGGG, respectively. Bars = 20 μm. **J** and **L)** BiFC interactions between the 2 mutated DTT1 proteins with HvDYT1, **J)** DTT1-△1 and DYT1, **L)** DTT1-△2 and DYT1. Bars = 20 μm. **K** and **M)** In vitro protein Y2H interaction assay between mutated DTT1 proteins with HvDYT1, **K)** DTT1-△1 and DYT1, **M)** DTT1-△2 and DYT1. Both **L** and **M** confirm the interaction between the mutated DTT1-△2 proteins and DYT1. Negative controls of DTT1-△1 **G)** and DTT1-△2 **I)** with empty pEXP-AD vector. **N)** All Y2H interactions and controls with serial dilution on nonselective media (SD-Leu), selective media (SD-LTH), and selective media with 3AT (SD-LTH 15 mm 3AT), showing no self-activation under selective conditions. +/++, indicates level of yeast survival indicating interaction on plates.

Previous studies showed that bHLH TFs are predominantly expressed in nuclei, but in some cases, such as AtDYT1 and PIF proteins, they are expressed in both cytoplasm and nuclei but can be relocated into the nuclei by interacting with other proteins (Pfeiffer et al. 2012; Cui et al. 2016). We examined the subcellular localization of barley DTT1 and HvDYT1 via transient expression in *Nicotiana benthamiana*. The results indicated nuclear localization, with colocalization with 4′,6-diamidino-2-phenylindole nuclear staining (Supplementary Fig. S5). Forming a complex with itself or other bHLH proteins is critical for bHLH protein molecular function (Chapman-Smith and Whitelaw 2006). A single basic region from the bHLH domain cannot stably bind DNA, as the basic region requires the HLH region to form a homodimer or heterodimer to bring them together to effectively bind the target DNA (Chapman-Smith and Whitelaw 2006). No detectable self-interactions were seen for DTT1 protein in both in vivo and in vitro protein interaction assays (Fig. 5, A, B, and N). We then investigated the interaction between DTT1 and HvDYT1, the other key bHLH proteins in anther tapetum development (Fig. 5). Both in vivo and in vitro protein interaction assays confirmed the interaction between them (Fig. 5, C, D, and N).

When we conducted phylogenetic analysis of the bHLH genes from both monocot and dicot plants, the protein secondary structure indicated they all contain the ββαββα topologies (ACT-like(BIF) domain) at their C-terminal (Fig. 1A). Functional study of this domain in *Arabidopsis* DYT1 showed that it helped relocate AtDYT1 into tapetal cell nuclei through interacting with other bHLH proteins, and 4 amino acids, F139, L141, I143, and I144, were critical for protein complex formation (Cui et al. 2016). Protein sequence alignment for the ACT-like(BIF) domain from these reported anther bHLH proteins indicated that the DYT1 orthologs and these bHLH proteins share a highly conserved IKL motif; however, a unique KRRR motif is detected only from these bHLH89/91/10 and ortholog proteins (Fig. 5E). To verify the molecular function of these 2 motifs in DTT1, we mutated them to DQD and CGGG, respectively. These 2 mutated versions were termed as DTT1-delta1(△1) and DTT1-delta2(△2). Transient expression was firstly conducted to determine if the mutation would result in any changes in protein subcellular localization in the cell; the results showed that both mutated protein versions were fully nuclear localized (Fig. 5, F and H). There was not any detectable self-activation from these 2 mutated proteins in yeast prior to preforming both in vivo and in vitro protein interaction assays (Fig. 5, G and I). We subsequently conducted protein interaction assays between the mutant proteins and HvDYT1. No interaction could be detected between DYT1 and DTT1-△1, but the DTT1-△2 maintained interactions with DYT1, indicating that IKL amino acid residues are essential for forming complexes between 2 bHLH proteins (Fig. 5, J to N).

**Figure 6.**
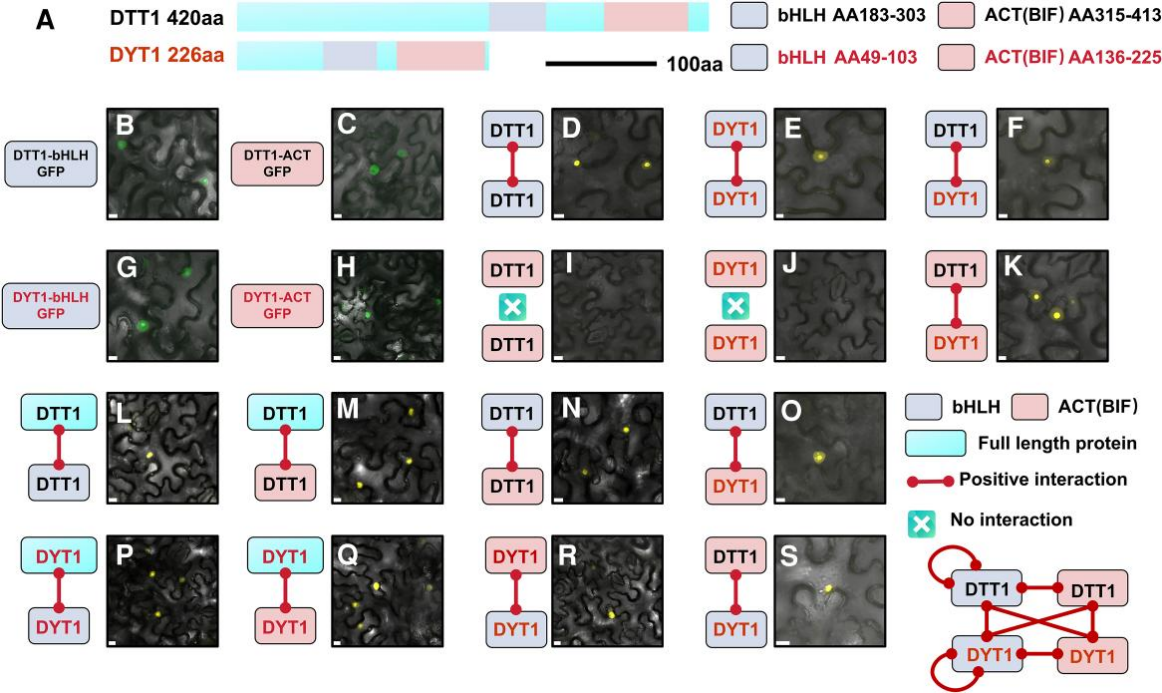
Verifying the key regions in the interaction between DTT1 and HvDYT1. **A)** Protein structures of DTT1 and HvDYT1, showing bHLH and ACT(BIF) domains, and the truncated protein sequences used to test these domains and their interactions. **B, C, G,** and **H)** Subcellular protein localization of truncated proteins tagged with GFP in *N. benthamiana* leaves. **B)** DTT1-bHLH, (C) DTT1-ACT-like(BIF), **G)** HvDYT1-bHLH, **H)** HvDYT1-ACT-like(BIF). Bars = 25 µm. **D, E,** and **I–S)** BiFC interactions between full-length protein, bHLH domain, and ACT(BIF) domain in DTT1 and DYT1. YFP signal indicates protein–protein interaction. **D–F)** BiFC interactions between homo- and hetero-bHLH domains. **I–K)** BiFC interactions between homo- and hetero-ACT-like(BIF) domain. **L** and **M)** Interaction between full-length DTT1 with its bHLH and ACT-like(BIF) domain. **P** and **Q)** Interaction between full-length DYT1 and its bHLH and ACT-like(BIF) domain. **N, O, R,** and **S)** Interaction between different bHLH and ACT-like(BIF) domains. Schematic illustrates the interactions between different truncated domains. Solid lines are positive interactions, and crosses are negative interactions. Bars = 10 µm in B–D, H–J, L, M, and P–S. Bars = 7.5 µm in F, G, N, O, and Q. Bars = 5 µm in E and K.

To comprehensively investigate potential interaction regions in DTT1 and DYT1, we performed systematic protein truncation analyses (Fig. 6A). Specifically, both bHLH and ACT-like(BIF) domains were incorporated into the BiFC system to assess homo- and hetero-protein interactions. Prior to BiFC experiments, we determined the subcellular localization patterns of these truncated domains by fusing them with SV40-NLS and a C-terminal GFP reporter. Both bHLH and ACT-like domains from DYT1 and DTT1 exhibited nuclear localization (Fig. 6, B, C, G, and H). We further verified the homo- and hetero-interactions between bHLH domains using BiFC, which revealed distinct interaction patterns. Both DTT1 and DYT1 demonstrated both homotypic and heterotypic bHLH domain interactions (Fig. 6, D to F), while ACT-like(BIF) domains exclusively showed heterotypic interactions without detectable homotypic interactions (Fig. 6, I to K). These findings suggest that DTT1 and HvDYT1 form heterodimers through dual-domain interactions, with the ACT-like domain potentially serving as the primary determinant in partner selection. Further investigation between the interaction of full-length proteins with their truncated proteins showed that both full-length DTT1 and DYT1 interacted with their truncated bHLH domains, producing nuclear signals comparable to those observed with truncated versions (Fig. 6, L and P). Contrary to expectations, ACT-like(BIF) domains maintained detectable interactions with their full-length proteins, a result inconsistent with truncated domain combinations (Fig. 6, M and Q). We further validated the positive interaction between the bHLH and ACT-like(BIF) domain from either the same protein or 2 different proteins (Fig. 6, N, O, R and S). These findings suggested that monomeric, bHLH proteins may assemble into alternative complex configurations distinct from conventional bHLH heterodimers.

Interestingly, most bHLH proteins have been reported to show self-interaction ability; in *Arabidopsis* DYT1, bHLH010, bHLH089, and bHLH091 all form homodimer complexes (Zhu et al. 2015). DTT1 is a putative paralog of bHLH010–bHLH091, but no self-interaction was detectable from both our in vivo and in vitro protein interaction assays. Previous reports of biomolecular complementation (BiFC) vectors use cauliflower mosaic virus35S promoter-based systems, whereas we used the *Arabidopsis* ubiquitin-10 (*AtUBQ10*) promoter-based system. The *AtUBQ10* system gives a lower protein expression level, which may explain why no self-interaction was detectable in our assay. We subsequently generated a set of CaMV35S promoter-based BiFC system with split YFP as reporter. The protein expression level test was done between DTT1–HvDYT1 combinations. An obviously increased signal density was observed when the *AtUBQ10* promoter was changed to the CaMV35S promoter (Supplementary Fig. S6, A to C). With increased protein level, self-interaction of the DTT1 protein could also be detected, and interaction between the mutated delta-1 version and HvDYT1 protein (Supplementary Fig. S6, D to F), which was not observed using *AtUBQ10* promoter (Fig. 5, A and J). These experiments indicate the important role of protein expression level in complex formation and show that DTT1 tends to form complexes with other bHLH proteins rather than itself under physiologically normal expression levels.

### DTT1 is an initiator for anther tapetum developmental transition from stage 6 and afterwards

**Figure 7.**
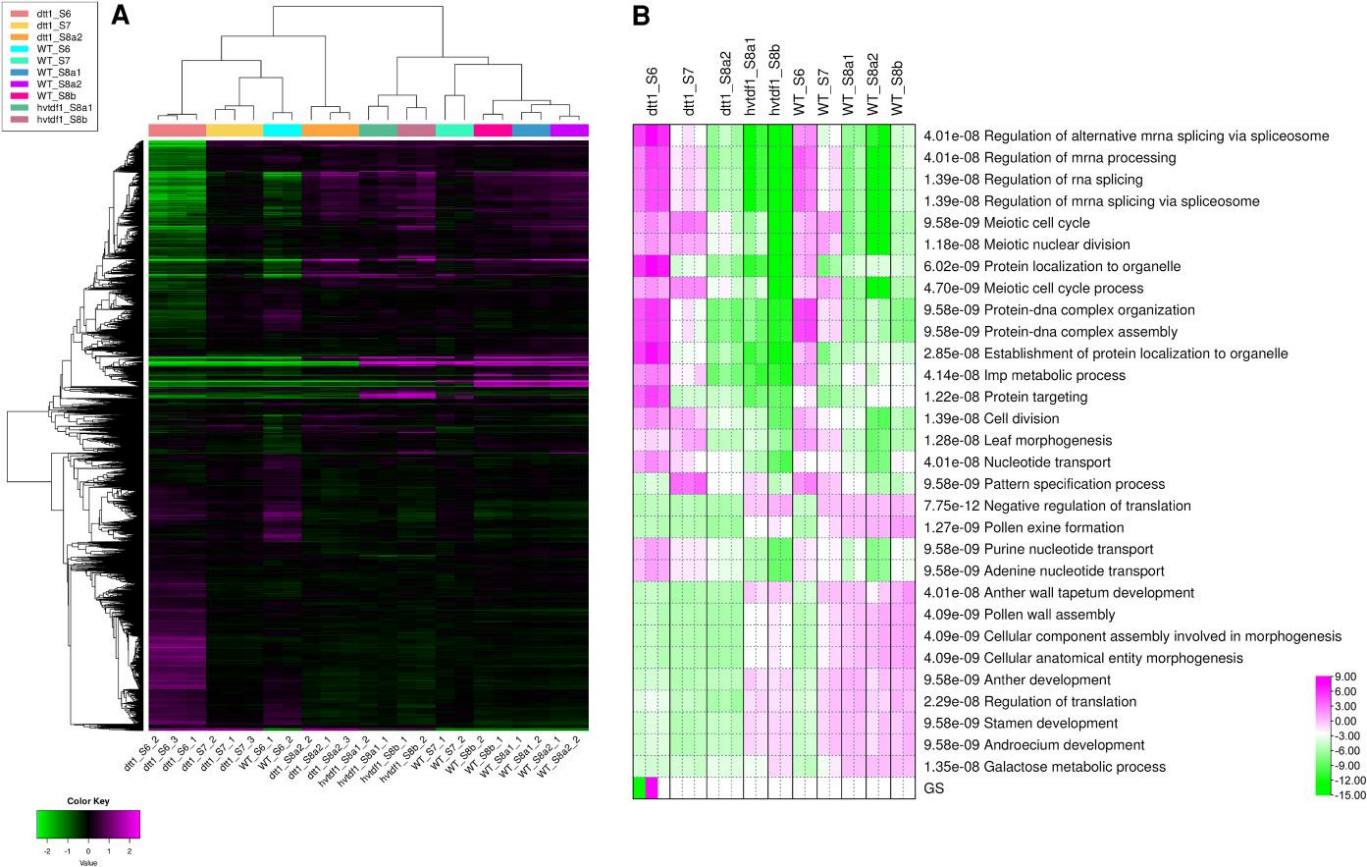
Transcriptome analysis between *dtt1-1*, *Hvtdf1-2* mutant, and wild type. **A)** Hierarchical clustering heatmap analysis among the 5 different developmental stages in wild-type, *dtt1-1*, and *Hvtdf1-2* mutants performed using IDEP.95 online RNA-seq platform conducted with standard deviation, showing the top-ranked 12,000 expressed genes across all samples. The color scale indicates the number of standard deviations from the overall average of expression (pink and green colors representing the activated and repressed patterns, respectively). The figure was generated using IDEP.95 online RNA-seq platform. **B)** Heatmap of biological process grouping among the 5 different developmental stages in wild-type, *dtt1-1*, and *Hvtdf1-2* mutants, based on GO terms conducted with PGSEA package (pink and green colors representing the activated and repressed patterns based on analysis data, respectively). Pathway significance cutoff (FDR < 0.05) and the top 30 pathways were presented on the heatmap. Pink and green indicate relatively activated and suppressed pathways, respectively. GS indicated the heatmap color scale. The color bar represents the activation and repression levels.

To determine the biological role of DTT1 in anther tapetum development, transcriptome comparison between wild type, *dtt1-1*, and *Hvtdf1* mutant was conducted. Based on the semi-thin section results, *dtt1-1* mutant spikes from stage 6, stage 7, and stage 8a2 were selected and sequenced with 3 biological replicates. Wild-type samples from stage 6 to stage 8b and *Hvtdf1* samples from stage 8a1 to stage 8b from our previous study were used (*dtt1* mutant samples were collected and sequenced at the same time as *Hvtdf1-2* and wild type) (Hua et al. 2023). Both principal component analysis (PCA) and correlation matrix showed strong correlations for each biological replicate (Supplementary Fig. S7, A and B). Hierarchical cluster analysis showed that all *dtt1* mutant stages grouped into the same clade as wild-type stage 6, suggesting that *dtt1-1* mutant failed to enter stage 7 (Fig. 7A). We also conducted *K*-means cluster analysis on these 12,000 genes, which were classified into 4 clusters (Supplementary Fig. S8A; Supplementary Table S1). The genes in cluster 1 demonstrated enrichment related to pollen and anther tapetum development, showing that most genes were downregulated in the *dtt1-1* mutant but only partially in the *Hvtdf1-2* mutant (Supplementary Fig. S8, A and B). To understand the overall pathways affected in *dtt1-1*, we conducted parametric gene set enrichment analysis (PGSEA). Most pathways from *dtt1-1* stage 6 showed a similar pattern as wild-type stage 6, suggesting gene initiation at stage 6 was not affected, but most stages related to anther tapetum, and pollen exine development did not progress through the transition from stage 6 to stage 7 (Fig. 7B; Supplementary Fig. S9, A and B). However, in *Hvtdf1*, these pathways showed downregulation rather than failure, which is consistent with our *HvTDF1* study (Fig. 7B; Supplementary Fig. S9) (Hua et al. 2023). This consequently resulted in most pathways relating to pollen development failing in the mutant at the following developmental stages (Fig. 7B). All these data support the critical role of DTT1 in anther transition from stage 6 to stage 7.

We conducted differentially expressed gene analysis with DESeq2 to identify putative regulatory targets for DTT1. We initially used a strict screen filter (false discovery rate [FDR] < 0.05 and fold change [FC] < −4), which identified 1,307, 438, and 781 downregulated genes from *dtt1-1* mutant stage 6, stage 7, and stage 8a2, respectively (Supplementary Table S1). The GO term analysis of these genes revealed that stage 7 and stage 8a1 are highly correlated with anther tapetum development and pollen wall material formation, whereas stage 6 is more related to the secondary metabolism process (Supplementary Fig. S10, A to C). A total of 324 genes are shared among stage 6, stage 7, and stage 8a1 (Supplementary Fig. S10D). Among these genes, we found that our previously reported HvTDF1 and its direct targets, osmotin proteins, showed no expression in the *dtt1-1* mutant, suggesting a potential DTT1–HvTDF1–osmotin regulatory genetic pathway in barley tapetum development (Supplementary Fig. S11C). *DTT1* expression in the *Hvtdf1-2* mutant still occurred, but with only approximately 50% expression (Supplementary Fig. S11B). Based on expression patterns, *DTT1* was expressed earlier than *HvTDF1*, suggesting *DTT1* is upstream of *HvTDF1* and may be involved in regulating *HvTDF1* expression. Thus, the genes that also showed downregulation in the *Hvtdf1-2* mutant were not included as putative targets; this left 225 genes (Supplementary Fig. S12A; Supplementary Table S1). We annotated these genes and identified their orthologs in *Arabidopsis* and rice; the GO term analysis results showed that tapetum development and anther development-related processes were enriched (Supplementary Fig. S12B; Supplementary Table S2). These findings suggest that DTT1 involved biological processes that are necessary for the anther transition from stages 6 to 7, which may unlock the expression of tapetum genes and activate downstream pollen wall formation. Interestingly, 3 bHLH TFs, *HvAMS*, and 2 copies of the putative *HvEAT1* genes were identified (Supplementary Table S2). Based on our wild-type RNA-seq data, all 3 bHLH genes were preliminarily expressed at stage 6 and showed dramatic increases at stage 7 (Supplementary Fig. S1, A and B; Supplementary Fig. S11D). However, in *dtt1-1* mutant stage 7 and stage 8a2 samples, the expression levels of these 3 genes showed similar levels as in wild-type stage 6 (Supplementary Fig. S11, D, F, and G). This suggests that their initial expression is regulated by genes upstream of DTT1, while DTT1 is involved in regulating them during the anther transition from stages 6 to 7.

In addition to HvTDF1, several MYB TFs were also not expressed in the *dtt1-1* mutant at both stages (Supplementary Table S2). These differed from the bHLH proteins, as these MYB genes were not expressed at stage 6 but were expressed later at stage 7. Interestingly, 2 of these MYB genes, HORVU.MOREX.r2.6HG0469220 and HORVU.MOREX.r2.7HG0544570, were annotated as putative orthologs of GAMYB, AtMYB65 and AtMYB101, in Arabidopsis (Supplementary Table S2). Their putative orthologs in rice are Os06g0679400 and OsGAMYBL2. Among these rice GAMYBs, Os06g0679400 was not previously annotated as a GAMYB-like protein; however, when we used its amino acid sequence against the *Arabidopsis* database, it showed high similarity to AtMYB101, termed as OsGAMYBL3. This finding suggests that there may be additional unverified putative GAMYB-like proteins in barley. We then used all these GAMYB proteins against the barley genome and surprisingly identified the other homolog of HvGAMYB, termed HvGAMYB2; they showed similar expression patterns to each other and the candidate ortholog of OsGAMYB, a key factor reported in rice tapetum development (Liu et al. 2010). HORVU.MOREX.r2.6HG0469220 and HORVU.MOREX.r2.7HG0544570 were termed as HvGAMYBL1 and HvGAMYBL2, respectively. Phylogenetic analysis was conducted to understand the evolutionary relationship between the GAMYB proteins in these 3 species; this showed that both HvGAMYB and HvGAMYB2 have a close relationship with OsGAMYB and AtMYB33/AtMYB65 (Millar and Gubler 2005; Liu et al. 2010); the other barley GAMYBs were grouped into subclades with AtMYB101 (Supplementary Fig. S13). Previously, we showed that HvGAMYB was not affected in the *Hvtdf1-2* mutant (Hua et al. 2023); here, its expression level also showed no significant difference in the *dtt1-1* mutant (Supplementary Fig. S11H). The homolog HvGAMYB2 showed a similar expression level as HvGAMYB, which was not affected in either *Hvtdf1-2* or *dtt1-1* mutants (Supplementary Fig. S11I). These results suggest there may be a parallel relationship between these additional bHLH proteins and HvGAMYB1/2 to determine tapetum development in barley. The downregulation of HvGAMYBL1 and HvGAMYBL2 in *dtt1-1*, but not in *Hvdtf1-2*, mutant suggests a potential regulatory relationship between DTT1 and HvGAMYB-L1/L2 proteins from stage 6 to stage 7 (Supplementary Fig. S11, J and K). The remaining genes belong to P450 proteins, GDSL-like lipase (Supplementary Table S2); the barley *Dcl5* ortholog was included, which suggests that the *bHLH-Dcl5-24nt phasiRNA* pathway may also be conserved in barley (Supplementary Fig. S11M). The latest study in rice has revealed that 2 β-(1,3)-galactosyltransferases, UPEX1 and UPEX2, serve as direct targets of the UDT1 (DTY1 ortholog)–TIP2 (DTT1 ortholog) protein complex, which appears essential for maintaining tapetal secretory functions (Wang et al. 2025). We also identified their barley orthologs, HORVU.MOREX.r2.3HG0248470 and HORVU.MOREX.r2.1HG0053490, within the 225 downregulated genes, indicating evolutionary conservation of this regulatory module. Some of these, such as root cap proteins and proton-dependent oligopeptide transporter (POT) family proteins, were identified in this study and may be involved in anther development transition from stages 6 to 7. There were also some genes previously reported as downstream genes of AMS in *Arabidopsis*, such as ABC-2 type transporters (ABCG23) and NADPH oxidase (RBOHE) (Supplementary Fig. S11, N and O) (Xu et al. 2010; Xie et al. 2014).

### The formation of the DTT1 complex is necessary for DNA binding and regulating the expression of target genes

**Figure 8.**
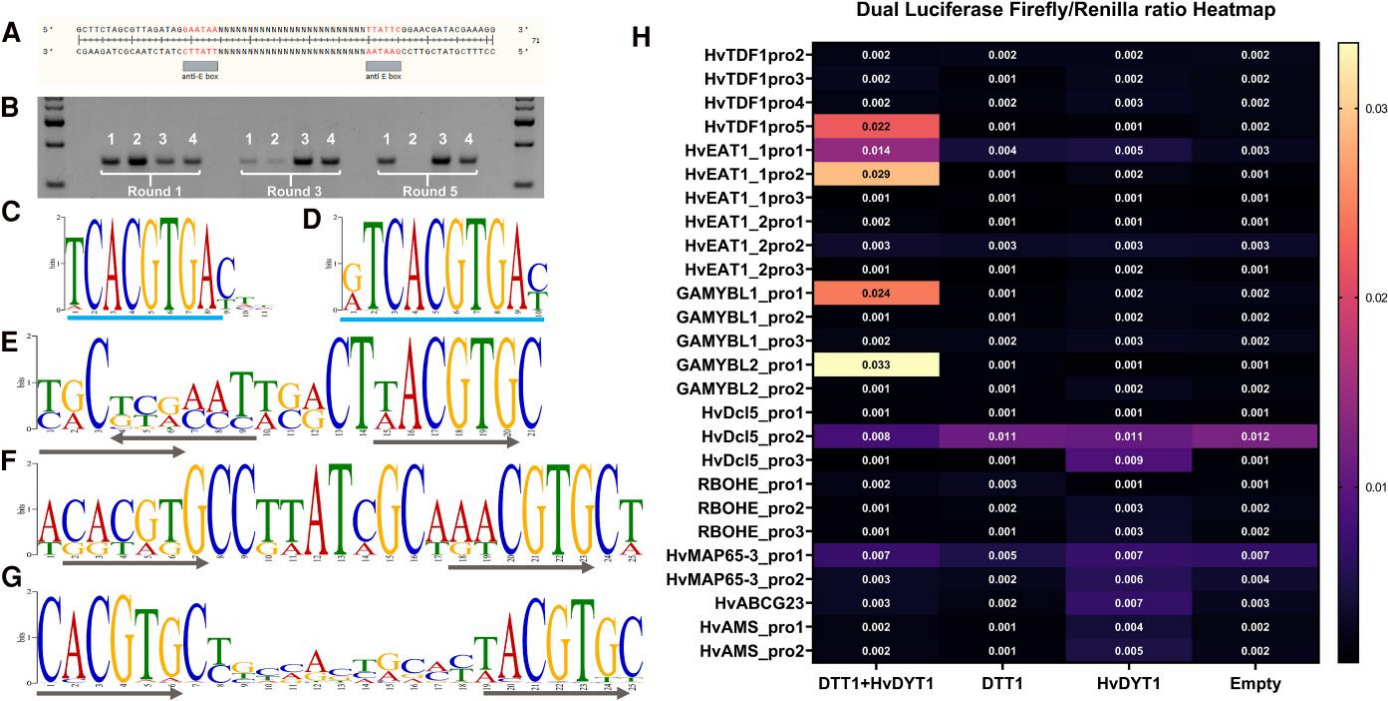
SELEX-seq assay determined the binding ability of the DTT1–DYT1 complex and validated regulating targets of DTT1–HvDYT1 by the dual-luciferase assay. **A)** Artificial DNA library with a 25-bp degeneration region flanked with anti-E-box region. **B)** Protein–DNA incubation enriched products from SELEX round 1, round 3, and round 5. Band 1 indicates the products from the DYT1 homodimer; band 2 indicates the products from the DTT1 homodimer; band 3 indicates the products from the DTT1–DYT1 heterodimer; band 4 indicates the products from the 3×HA negative control. **C** and **D)** Enriched motif of the HvDYT1 protein. **E, F** and **G)** Enriched motif of the DTT1–HvDYT1 complex; the arrows indicate orientations between the canonical E-box and noncanonical E-box. **H)** Dual-luciferase results showing positive interactions with HvTDF1, HvEAT1, and GaMYBL1/2 with DTT1/HvDYT1 heterodimer.

bHLH proteins generally show binding affinity to the E-box DNA motif, 5′-CANNTG-3′. We conducted a SELEX-seq assay to assess the DNA binding and enrichment capabilities of DTT1 and HvDYT1 individually, as well as the complex formed by their interactions. A DNA library with 25-bp degenerated region in the middle region plus the 6-bp anti-E-box motif on the flanking regions was prepared (Fig. 8A). We conducted SELEX with a total of 6 rounds of enrichment and 4 different proteins, HvDYT1, DTT1, DYT1–DTT1 complex, and negative control. The eluted DNA samples from the 1st, 3rd, and 5th rounds were amplified with universal primers. The agarose gel electrophoresis results showed that HvDYT1, coexpressed DTT1–DYT1, and the negative control can bind and enrich DNA from each round (Fig. 8B). DTT1 on its own had less DNA binding affinity compared with other proteins tested, with no band detectable in the 5th round (Fig. 8B). Sequencing was used to identify the DNA sequences from 6th-round eluted samples with at least 50,000 reads for each sample. We initially tried to identify DNA binding motif from HvDYT1; 8 bp, TCACGTGA, and 10 bp, G/ATCACGTGAC/T, 2 palindromic E-box motifs were enriched, which is consistent with the reported *Arabidopsis* DYT1 binding motif (Fig. 8, C and D) (Feng et al. 2012). This finding also suggests that DYT1 might also be conserved between monocot and dicot plants. No obvious E-box-related motifs were enriched from the negative control (Supplementary Fig. S14, A and B). We then detected the motifs from DTT1–HvDYT1 complex eluted sample. Most enriched motifs showed a mixed E-box motif, CACGTG, linked with the other E-box-like motif TA/TCGTG (Fig. 8E). Analysis of the sequences from the original raw sequencing data indicated that the canonical E-box showed a mixed pattern with 2 different orientations and variant space between 2 motifs (Fig. 8, F and G; Supplementary Fig. S15, A to D). We also determined that the DTT1–HvDYT1 complex showed an activation role in regulating gene expression through the modified dual-luciferase assay, in which the HvDYT1 protein itself did not show activation ability (Supplementary Fig. S16). Subsequent analysis of putative regulating targets from RNA-seq data revealed that the DTT1–HvDYT1 complex is capable of regulating the expression of HvTDF1, HvGAMYBL1, HvGAMYBL2, and HvEAT1-L1 (Fig. 8H, Supplementary Fig. S17).

## Discussion

### DTT1 may serve as a part of a paired key to unlock tapetal cell transition initiation

During early anther development, the PPC layer undergoes an anticlinal division to form 2 SPC layers. The outer SPC layer enters a further periclinal division and differentiates into the endothecium. The inner one undergoes a new round of anticlinal divisions to form the middle layer and tapetum. This establishes the 4 anther somatic cell layers that surround the PMCs. The middle layer becomes thinner and initiates degeneration at anther stage 7. However, the tapetum requires further differentiation to acquire its specific cell fate, through endomitosis to form 2 nuclei in each tapetal cell. The tapetum cell wall subsequently degenerates and is replaced with Ubisch bodies, spur-like structures on the outer surface face of the developing microspores. The differentiated tapetum then regulates the PMC meiosis process, callose breakdown and microspore release from tetrads, pollen wall precursor formation, and pollen coat protein synthesis for immature pollen grain development (Shi et al. 2015).

In the *dtt1-1* mutant, both tapetum endomitosis and cell wall degeneration fail (Figs. 4, E to H; and 3, S to V). Expression hierarchical clustering showed that mutant stage 7 and stage 8a2 grouped into the same subclade as wild-type stage 6 (Fig. 7A). The PGSEA also showed that the enriched pathways of *dtt1-1* stage 7 are similar to wild-type stage 6, and most failed in the following stage 8a2 (Fig. 7B; Supplementary Fig. S9). The DEGs from each stage revealed a total of 324 downregulated genes across the different stages (Supplementary Fig. S10). We found that some previously reported tapetum development key TFs, such as *HvTDF1* and *HvAMS*, were not expressed or expressed only at extremely low levels in *dtt1-1*. *HvTDF1* ranked as the highest log2FC value within the identified genes, and *HvAMS* showed expression at the preliminary expression level as seen in wild-type stage 6. In our previous *HvTDF1* studies, *HvAMS* remained at almost half the normal expression level in the *Hvtdf1-2* mutant, and HvTDF1 showed relatively weak activation of *HvAMS* expression compared with other direct targets, such as *OSMOTIN1/2/3* (Hua et al. 2023). This confirmed our previous hypothesis that the relationship between *HvTDF1* and *HvAMS* is not a simple linear regulatory pathway, and other genes may be involved in activating *HvAMS*. Here, we have verified that the activation of *HvTDF1* expression is dependent on DTT1–HvDYT1 complex formation (Fig. 8H). However, there is a difference compared to the *Arabidopsis* gene network. The expression level of *AtTDF1* was not affected in the *bhlh010 bhlh089 bhlh091* triple mutant and only showed downregulation in the *Atdyt1* mutant, which suggests that the complex between bHLH and DYT1 may not be necessary to regulate *AtTDF1* expression (Gu et al. 2014; Zhu et al. 2015). This suggests a divergence between dicot and monocot plants among these bHLH proteins.

To understand the biological role of DTT1 in tapetum transition from stages 6 to 7, we utilized our *Hvtdf1-2* transcriptome data, since in *Hvtdf1-2*, both tapetum endomitosis and cell degeneration were delayed rather than abolished as seen in *dtt1-1* (Hua et al. 2023). We found that, among the 324 downregulated genes, 225 genes are not changed in *Hvtdf1-2* (Supplementary Fig. S12). The remaining genes also showed downregulation in *Hvtdf1-2*, such as *OSMOTIN1/2/3*. These 225 genes may therefore play important roles during the tapetum transition process; included in this group were the remaining 2 barley bHLH proteins, HvEAT1-L1 and HvEAT1-L2, suggesting they may be functionally redundant; the copy numbers of these are different compared with other monocot plants, such as rice and maize (Niu et al. 2013; Fu et al. 2014; Nan et al. 2016). Several copy number differences have been reported for these tapetum bHLH TFs; in the dicot *Medicago truncatula*, there are 2 anther bHLH genes, *EMPTY ANTHER1 (EAN1)* and *EAN2* (orthologs of *Arabidopsis bHLH010*, *bHLH089*, and *bHLH091*), with *EAN1* rescuing the fertility in *bhlh010 bhlh089* double mutant (Zheng et al. 2020). There are also 2 copies of *AMS*, *MtAMS* and *MtAMSL2*, in *M. truncatula* compared with previous reports in monocot or dicot plants (Zheng et al. 2020). The variation in copy numbers of these additional tapetum bHLH genes in barley is probably due to the shorter domestication period and more gene duplication events compared with rice and maize. Thus, we have established that in barley, there are 3 bHLH proteins, HvAMS, HvEAT1-L1, and HvEAT1-L2, which are expressed during the anther tapetum transition from stages 6 to 7. DTT1 and HvDYT1 show a relatively prolonged expression pattern over this developmental period. Given their ability to dimerize, there are several putative pairwise combinations among these bHLH proteins that might serve as “paired keys,” which may be responsible for unlocking numerous specific biological processes.

### DTT1 works as an announcer for tapetum and meiocyte development

The highly conserved tapetum regulatory genetic pathway, *DYT1–TDF1–AMS–MYB80–MS1*, has been characterized in both monocot and dicot plants. However, evidence indicates that this genetic pathway includes several feedforward and feedback loops, which are influenced by additional anther-specific bHLH proteins. There are also some key transcription factors characterized in *Arabidopsis* and rice, such as GAMYB proteins, which were identified in our transcriptomic analysis. We found 2 GAMYB proteins, HvGAMYB and HvGAMYB2, the putative orthologs of the rice and *Arabidopsis* genes; their expression was also not affected in *dtt1-1*, suggesting that the relationship between DTT1 and GAMYBs may be conserved in these species. However, 2 additional stage-specific GAMYB-like proteins, HvGAMYB-L1 and HvGAMYB-L2, were also identified (Supplementary Fig. S13). We found that the expression of these 2 GAMYB-like proteins could be activated by the DTT1–HvDYT1 complex (Fig. 8H). An additional gibberellin-regulated protein was also identified. These findings indicate that GA signal-related pathways involved in early anther development are working in parallel and overlap with DTT1-related pathways.

Previous studies in rice and maize showed that bHLH TF genetic pathways are important for PMC meiosis by regulating *Dcl5* (previously called *Dcl3b* in rice) to produce 24-nt phasiRNAs to control meiosis-associated gene expression (Ono et al. 2018; Nan et al. 2022). However, control of *Dcl15* expression was different between the 2 species, for example, through Ms32 (UDT1 and DYT1) and Ms23 (TIP2) complex and bHLH122 (EAT1) in maize (Nan et al. 2022). Nevertheless, Dcl5 expression relies on both EAT1–TDR and UDT1–TIP2 complexes (Ono et al. 2018). Interestingly, orthologs of these bHLH were also reported in *Arabidopsis*, but no 24-nt phasiRNAs were reported in *Brassicaceae*, suggesting a different mechanism of *Arabidopsis* DYT1-bHLH010/089/091 regulation (Xia et al. 2019; Nan et al. 2022). *HvDcl5* showed less expression in *dtt1-1* in contrast to *Hvtdf1-2*, suggesting that its expression also requires DTT1-related pathways, for normal anther transition. We also investigated the potential regulatory relationship between the DTT1–HvDYT1 complex and *HvDcl5*, but no activating role was confirmed. This suggests that *HvDcl5* expression is likely regulated by other currently uncharacterized genes in barley.

**Figure 9.**
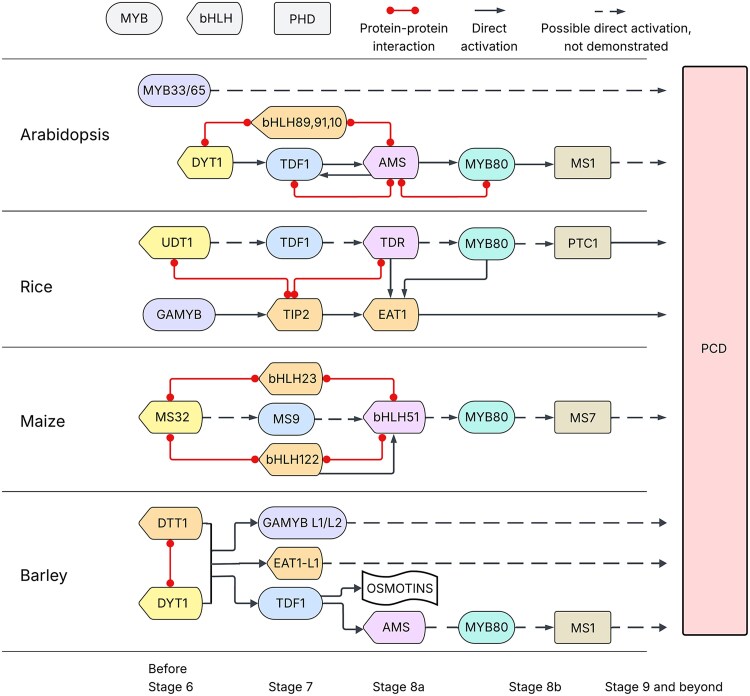
The putative regulatory pathway in barley tapetum development, in comparison to other species. Color of box edges links orthologs across species. In barley, DTT1 acts upstream of HvTDF1 and works together with HvDYT1 to initiate the tapetum transition process by activating stage 7-specific expressed TFs. Arrows indicate direct activation, dots indicate protein–protein interaction, and dotted arrows indicate activation that has not yet been experimentally proven as direct; PCD, programmed cell death.

The tapetum serves as a nurse tissue and requires precise cell fate determination to establish its secretory capacity for providing critical developmental substrates and enzymatic components for PMC development. Recent study in rice has demonstrated that 2 β-(1,3)-galactosyltransferases, UPEX1 and UPEX2, serve as direct regulatory targets of the UDT1–TIP2 protein complex, playing indispensable roles in this developmental cascade (Wang et al. 2025). Notably, functional conservation of *UPEX* homologs has been observed across both monocot and dicot plants, suggesting their evolutionary significance in tapetal development. Our transcriptomic analysis also revealed that the barley orthologs *HvUPEX1* and *HvUPEX2* were identified within the 225 downregulated genes (Supplementary Table S2), reinforcing the phylogenetic preservation of this regulatory module. The other downregulated genes belong to sugar, lipid transporter, and P450 subfamilies, suggesting the transition from stages 6 to 7 requires high amounts of energy and active metabolism. There are several MYB proteins with specific expression from stage 6 to stage 7, suggesting this transition may require numerous regulatory pathways working together. These studies, focused on both *DTT1* and *HvTDF1,* have established the gene network of tapetum development in barley, which comprises both direct and indirect regulation involved in this tapetum determination transition (Fig. 9).

### The ACT-like(BIF) domain may play a selective role in bHLH protein interactions

Functional bHLH proteins need to form homodimers or heterodimers to regulate gene expression (Amoutzias et al. 2004). Dimerization is normally achieved through the HLH motif of the bHLH domain; the basic regions from each bHLH protein contribute to the DNA binding (Brownlie et al. 1997; Amoutzias et al. 2004). However, there are also other additional domains that help stabilize the homodimers or heterodimers, such as the leucine zipper, Per-ARNT-Sim (PAS), or “Orange” domains at the C-terminal region (Ledent and Vervoort 2001). Apart from structure stabilization, the bHLH C-terminal domains also show an important role in selecting interaction partners among bHLH proteins. Both aryl hydrocarbon receptor (AhR) and aryl hydrocarbon receptor nuclear translocator (Arnt) belong to the bHLH-PAS proteins and can form heterodimers (Whitelaw et al. 1993; Isaacs et al. 2004; Lee et al. 2004; Schmid et al. 2007; Inamoto et al. 2017). The truncated bHLH domain from AhR was able to form homodimers and heterodimers with full-length Arnt, which was sufficient to bind regulatory targets (Pongratz et al. 1998; Chapman-Smith and Whitelaw 2006; Inamoto et al. 2017). AhR bHLH domain fused with the partial N-terminal portion of the PAS domain failed to form homodimers but was still able to form heterodimers with Arnt (Hao et al. 2011), indicating that PAS domains have a role in partner selection.

In plants, the first characterized bHLH TF, R, cooperates with the R2R3 MYB family gene, C1, to regulate anthocyanin biosynthetic gene expression in maize tissues (Ludwig and Wessler 1990). R comprises an N-terminal MYB-interacting region and a central bHLH domain, followed by a C-terminal ACT-like domain (Ludwig and Wessler 1990; Feller et al. 2006). The ACT-like domain showed similar structure to the ACT domain, which was found in many metabolic enzymes (Feller et al. 2006). Evolution studies suggest that the C-terminal domain in bHLH proteins resulted from a protein fusion between bHLH and ancestor ACR (ACT DOMAIN REPEAT) gene (Lee et al. 2023). The ACT-like domain was reported as a specific topology, ββαββα, at the C-terminal of numerous plant bHLH proteins (Feller et al. 2006). The ACT-like domain in maize R protein acts as a dimerization domain with 2 pathways: R protein forming a homodimer under the “ON” model to bind E-box motif and a C1-RIF1-R complex with “OFF” to regulate maize A1 gene expression (Kong et al. 2012). In anther development, this topology was also identified in *Arabidopsis* tapetum-specific bHLH gene, DYT1, which was termed as the bHLH protein interaction and function (BIF) domain (Cui et al. 2016). The BIF domain contributes to form heterodimers with bHLH089 and relocalizes the complex into nuclei to activate TDF1 expression.

Here, we also identified an ACT-like (BIF) domain at the C-terminal of the barley DTT1 protein (Fig. 1A). We found the DTT1 was not able to homodimerize in both BiFC and Y2H assays (Fig. 5, A and B) but interacted with HvDYT1 (Fig. 5, C and D). The highly conserved IKL motif of DTT1 ACT-like (BIF) domain was confirmed as important for DTT1–HvDYT1 complex formation (Fig. 5, J and K). However, when we compared our protein–protein interaction assays, the promoter we used (*AtUBQ10*) gave a relatively lower expression reflecting normal tapetum expression levels compared to other published assays (Cui et al. 2016). We were only able to observe DTT1 homodimerization when we used a high-level expression system (CaMV35S promoter) (Supplementary Fig. S6D). Using truncated bHLH and ACT-like domains in our low (“native-level”) expression BiFC system we were able to see that the bHLH domain from both proteins can form either homo- or hetero-interactions, but the ACT-like (BIF) domain can only form hetero-interactions (Fig. 6, J to L). These results demonstrate that DTT1 protein tends to form heterodimers rather than homodimers, and the ACT-like domain may play the critical role in partner selection.

### DTT1 may modulate target gene expression through dual E-box motifs by forming a higher-order complex with DYT1

The SELEX results showed that both HvDYT1 and DTT1–HvDYT1 complex could enrich the fragments, but not for DTT1 (Fig. 8B). Studies of maize R protein showed that its DNA binding ability was abolished by the homodimerized C-terminal ACT-like domain, and that E-box binding only occurred when the monomeric ACT-like domain was present (Kong et al. 2012). It is clear that the palindromic E-box motif, TCACGTGA, was enriched by HvDYT1, which is consistent with previous reports for its ortholog gene, AtDYT1, in Arabidopsis (Fig. 8, C and D) (Feng et al. 2012). However, the motif enriched from DTT1–HvDYT1 complex showed a partly hidden linked Dual E-box pattern, which consisted of both canonical and noncanonical E-box motifs (Fig. 8, E to G). After checking the original raw sequencing data of DTT1–HvDYT1, we found that the orientation between these 2 E-box kept in both the same and reversed orientation at 1 site, which thereby resulted in the chimeric pattern for the canonical E-box. The noncanonical E-box (TACGTG) motif has been reported on Hypoxia-Response Element promoter and directly regulated by hypoxia-inducible factors (HIFs) (Kimura et al. 2001). The HIFs form the bHLH homodimer and bind to the canonical E-box, CACGTG, flanked with T and A (TCACGTGA) also found in HvDYT1 SELEX enrichment. When alpha and beta subunits form a heterodimer, this complex recognized noncanonical TACGTG (5′-RCGTG-3′, R is A or G) motif (Chen et al. 2022). The pairwise combinations among HIF alpha and beta subunit also showed the specific binding to NNCGTG motif (Bersten et al. 2022).

Although the HIF-related motif has not been reported in plants, hypoxia is a common physiological phenomenon seen in plants, which has been classified as acute and chronic (Weits et al. 2020); acute hypoxia is a result of environment stress, such as flooding, whilst chronic hypoxia means the low oxygen condition within specific region of plant body by locally high respiration rates or barriers that limits oxygen diffusion between cells (Weits et al. 2020). Chronic hypoxia has been linked to developmental regulation, such as shoot apical meristem (SAM) activity, lateral root transition, pollen germline cell fate (Kelliher and Walbot 2012; Meitha et al. 2015, 2018; Shukla et al. 2019; Weits et al. 2019). The mechanism shares similarity between mammals and plants, key HIF TFs, or ERF-VII are degraded through ubiquitination under normoxia but stabilized and relocated into nuclei, regulating downstream hypoxia-responsive element (HRE) expression under hypoxic condition (Ruas and Poellinger 2005; Schofield and Ratcliffe 2005; Dunwoodie 2009; Gibbs et al. 2011; Licausi et al. 2011). In maize male germline defective mutant, *msca1* (no germline cells), exogenously applied hypoxia could stimulate germ cell formation and rescue germinal cell differentiation (Kelliher and Walbot 2012) indicating a potential relationship between chronic hypoxia condition and anther cell development.

During anther development, ROS accumulation can be detected in both middle layer and endothecium from stage 5, which corresponds to the preliminary establishment of the 4 somatic cell layers (Yu and Zhang 2019). At stage 7, ROS signal is rarely detected in the tapetum but increases after this point (Yu and Zhang 2019). This suggests that tapetum cell fate acquisition is critical for activating downstream ROS accumulation genes at stage 7. ROS accumulation is considered a consequence of hypoxia, by reducing the aerobic oxidative respiration and affecting the electron-transport rate in mitochondria (Wang et al. 2007; Kondoh et al. 2013). We hypothesize that when all somatic cell layers are established in wild type, that the innermost tapetum layer may also be under hypoxia. The DTT1–HvDYT1 complex may therefore play a comparable role to HIFs, that regulate downstream gene expression through a similar motif (TACGTG) identified on HRE promoter, to ensure the tapetum cell fate transition.

The bHLH hetero- or homodimer binding to a dual E-box motif has not been previously described, but studies on mammalian circadian related genes, such as *period 2* (*Per 2*), showed a specific repeat E-box and E-box like pattern, with a current 6 bp space between them, which was required for its autonomous transcriptional oscillation expression pattern (Nakahata et al. 2008). Also, a coordinator formed between a single E-box and Homeodomain TF binding motif with a specific space is required in determining cell type in human face development (Kim et al. 2024). The Arabidopsis MYC2 protein, a MYC-type bHLH TF, binds to a Dual E-box motif by forming a homo-tetrameric complex, thereby inducing DNA looping to regulate gene expression (Lian et al. 2017). Our BiFC assays further revealed an interaction between the bHLH and ACT-like (BIF) domains, suggesting that 2 DTT1–HvDYT1 heterodimers may associate to form a higher-order complex, which could play a role in transcriptional regulation.

Combined together, our results indicate that even when the 4 somatic cell layers are established in barley anthers, the cell layers require further development to acquire their specific cell fate. The transition from anther development stage 6 to 7 is critical for normal function and initiation of downstream genetic pathways. At this point the tapetum requires multiple TFs working together, which may involve parallel networks with specific overlaps. Among them, the paired key formed by DTT1–HvDYT1 showed an activation role in announcing the initiation of this highly conserved tapetum regulatory genetic pathway through direct regulation of numerous TFs, such as HvTDF1, HvEAT1-L1 and GAMYBs. Alongside this, DTT1 or HvDYT1 may also be involved in other pathways through the ACT-like (BIF) domain partner selection ability. Thus, they could collectively perform a symphony to ensure normal anther and pollen development is tightly regulated and potentially adaptable under different environmental conditions.

## Materials and methods

### Barley growth condition and transformation

Two-row Spring barley cultivar, Golden Promise was used for transformation to generate CRISPR-CAS9 knock-out lines and as the wild-type control. Transgenic and wild type plants were grown under controlled growth conditions with 15 °C/12 °C; 16 h photoperiod; 80% RH, 500 µmol/m^2^/s metal halide lamps (HQI) supplemented with tungsten bulbs. The seeds were first sown in 12-well pots with John Innes No. 3 compost. After 2 to 3 wk, seedlings were transferred into 5-L pots with Levington CNSC compost (3 plants per pot); supplemented with 1 osmocote. CRISPR construct and barley Agrobacteria-mediated transformations were conducted according to Hua et al. (2023). Mutation detection of the target region was amplified with 6939F/6951R (Supplementary Table. S3).

### Molecular cloning of DTT1 and phylogenetic analysis

Bulked cDNA was prepared from different spike development stages and was utilized as a template to amplify the *DTT1* coding sequence by primer combination 7045F/7046R, designed based on the sequence HORVU.MOREX. r2.7HG0542700, with Phusion High-fidelity polymerase (Thermo Fisher) (Supplementary Table S3). The PCR amplicon of *DTT1* CDS was further confirmed by Sanger sequencing to determine the Single-Nucleotide Polymorphisms (SNP) between the transformation cultivar Golden Promise and the barley reference cultivar Morex. The confirmed DTT1 protein sequence was used to perform phylogenetic analysis with other orthologous genes by ClustalW and protein alignment by MEGA 11. A FASTA file of the alignment and a Newick file of the phylogenetic tree are provided as Supplementary Files 1 and 2. The protein secondary structure was predicted by https://www.predictprotein.org.

### Determining the expression pattern of *DTT1* transcript by RT-qPCR and RNA in situ hybridization

Total mRNA was extracted using the RNeasy Kit (QIAGEN, 74106) from different barley spikes sizes, representing different anther stages. For each sample (biological replicate), 3 *μ*g of total RNA was used to generate cDNA by Superscript IV VILO Master Mix with ezDNase Enzyme (Invitrogen 11766050). RT-qPCR was performed on a Roche LightCycler 480 Instrument II platform according to the following protocol: 95 °C for 2 min, 40 cycles of 95 °C for 5 s, and 60 °C for 35 s, 72 °C; Applied Biosystems PowerUp SYBR Green Master Mix (Thermo Fisher, A25776). Barley α-tubulin and Hsp70 were used as internal reference genes to normalize target gene expression level, with at least 2 biological replicates. RNA in situ hybridization was according to Fernández Gómez and Wilson (2014) with minor modification of in vitro RNA probe labeling and synthesis.

### Phenotypic analyses of the *dtt1* mutant

Plants and floral organs were photographed using a DMC-GX80 (Panasonic) and Zeiss Stemi 508, respectively. Floral samples for semi-thin sectioning were fixed with freshly made 4% (v/v) paraformaldehyde solution in 1.5 ml Eppendorf tubes. Samples were vacuum infiltrated for 30 min and then stored overnight at 5 °C. Samples were washed twice with 1× PBS buffer (Thermo Fisher Scientific, BP3991) and then dehydrated with increased ethyl alcohol series, 30%, 50%, 70%, 90%, and 100% (v:v), 1 h/step, except 100%, which was repeated twice. Samples were then embedded into Spurr resin (TAAB, medium hardness), according to the manufacturer's instructions; 1.5-μm transverse sections were cut with a Lecia EMUC7 ultramicrotome. For TEM sample preparation, freshly collected samples were fixed with 3% (v/v) EM-grade glutaraldehyde in 0.1 m cacodylate buffer and then treated with 1% (w/v) osmium tetroxide in 0.1 m cacodylate buffer. Washed samples were stained with 1% (w/v) aqueous uranyl acetate overnight, avoiding light to prevent precipitation of the uranyl acetate and then dehydrated through an ethanol series and embedded with TAAB Low Viscosity Resin (TAAB, T049; medium hardness), according to the manufacturer's instructions. The 50-nm ultrathin sections were then stained twice with 2% uranyl acetate and 2.6% (w/v) lead citrate aqueous solution. The samples were examined with the Tecnai G2 Spirit BioTwin transmission electron microscope (Tecnai) at 80 kV.

### Protein transient expression and bimolecular fluorescence complementation assay

The *DTT1* or *DYT1* full-length coding sequence or truncated versions without stop codons were PCR amplified and fused to the pUB-DEST-cYFP/nYFP for low expression level or p2× 35s-DEST-cYFP/nYFP by the Gateway cloning system. Sequencing confirmed vectors were cotransformed with pBIN-P19 into *Agrobacterium tumefaciens* strain GV3101. A positive *Agrobacterium* colony was incubated in liquid culture containing appropriate antibiotics overnight to reach an OD600 of 0.4 to 1. The liquid culture was then pelleted and resuspended in the infiltration buffer (10 mm MES, pH 5.8, 10 mm MgCl_2_, and 0.5 mm acetosyringone) to a final OD600 = 0.4. The different protein interaction combinations were prepared by combining the resuspended culture of nYFP- and cYFP-tagged proteins and infiltrated into 4 to 6 wk old *N. benthamiana* leaves. After 48 h, the interaction signal was observed under a Leica SP5 confocal microscope. This was repeated at least 3 times.

### Y2H analysis

The Y2H assay was performed using the Gateway cloning pDEST system (Invitrogen). *HvDYT1* and *DTT1* coding sequences were inserted into both pDEST32-BD and pDEST22-AD vectors, which were then transformed into Y187 and AH109 yeast strain based on the yeast heat shock PEG-based transformation method. Positive colony selection was conducted on SD-Leu and SD-Trp plates for pDEST32-BD and pDEST22-AD, respectively. The mating process was processed on YPDA plates; the first selection was then made on SD-Leu-Trp plates and then restreaked onto SD-His-Ade-Leu-Trp and X-α-Gal to test for positive interactions, which was repeated at least 2 times.

### TF activation assay and dual-luciferase assay

The modified dual-luciferase system was used to determine the TF function. The coding region was fused with the GAL4-DB domain, which was driven by the *AtUBQ10* promoter. In the dual-reporter vector, the original promoter insert region was replaced by the Gal4-UAS sequence, facilitating GAL4-DB-TF binding. These 2 vectors were transformed into GV3101; pUB-GAL4-DB-TF was cotransformed with pBIN-P19, while the pGreen800-GAL4-UAS-LUC was cotransformed with pSoup. The interaction protein, such as DTT1, was inserted into the pGWB5 vector, which was driven by the CaMV35S promoter, which was cotransformed into Gv3101 with pSoup. The liquid cultures of these 3 vectors were mixed and infiltrated into *N. benthamiana* leaves. After 2 days, luciferases were extracted from the infiltrated region using Passive Lysis buffer (Promega, E1941) according to the manufacturer's instructions. The Firefly and Renilla luciferases were analyzed using the Dual-Glo Luciferase Assay System (Promega, E2920) and detected by a Synergy LX Multi-Mode Reader following the manufacturer's instructions. To determine the direct activation of the DTT1–HvDYT1 complex, both coding regions of DTT1 and HvDYT1 were inserted into the pUB-DEST vector, respectively, to generate *AtUBQ10*pro-DTT1-GFP and *AtUBQ10*pro-HvDYT1 expression cassette and into the pGreen0802-mini35Spro-Luc reporter vector to generate 26 reporter vectors. The dual-luciferase assay was performed as above. For each combination, at least 6 biological replicates were tested.

### In vitro cell-free protein expression and SELEX-seq

Two vectors, pF3A-Kozak-3xHA-HvDYT1 and pF3A-Kozak-3xHA-HvDTT1, were generated for protein expression with TNT wheat germ cell-free protein system (Promega, L4380). The empty pF3A-Kozak-3xHA vector was used as the negative control. The anti E-box artificial dsDNA library was generated by annealing the ssDNA_AnitEbox_F and ssDNA_AnitEbox_R primers (Supplementary Table S3), which consist of a 20-bp degenerated region in the middle adjoined with the anti-E-box sequence on both sides. The SELEX-seq assays were performed as described by Smaczniak et al. (2017) with minor modifications on dsDNA library preparation. The enriched DNA were then amplified with 150bp-SELEX-F and 150bp-SELEX-R primer combination (Supplementary Table S3) to increase the PCR product size, which was sequenced on an Illumina HiSeq 2000 platform with 50,000 reads (GENEWIZ). The motif enrichment analysis was conducted using https://meme-suite.org/meme/tools/meme.

### Transcriptome sequencing and analysis

For RNA sequencing, single spikes were collected from single plants for each biological replicate. Spikes covered anther development stages 6 to 8a2 from *dtt1-1* mutants. The wild-type and *Hvtdf1-2 s*amples were collected alongside as described previously (Hua et al. 2023). Total RNA isolation, mRNA library preparation, and sequencing were conducted by a custom service (BGI; Hong Kong). Sequence data were mapped to the barley Golden Promise reference transcriptome and quantified by Galaxy quantification pipeline (Schreiber et al. 2020; Guo et al. 2021). Read numbers from each sample were used to generate the read matrix and analyzed by iDEP.95 (Ge et al. 2018). The BLAST barley genes to *Arabidopsis* followed the previously described approach (Hua et al. 2023).

### Accession numbers

Sequence data from this article can be found in the GenBank/EMBL data libraries under accession numbers: DTT1 (HORVU.MOREX.r2.7HG0542700), HvTDF1 (HORVU.MOREX.r2.4HG0319540), HvAMS (HORVU.MOREX.r2.6HG0456990), HvMYB80 (HORVU.MOREX.r2.2HG0144320), HvGAMYB (HORVU.MOREX.r2.3HG0246570), HvDYT1 (HORVU.MOREX.r2.2HG0112900), HvEAT1-L1 (HORVU.MOREX.r2.2HG0161050), HvEAT1-L2 (HORVU.MOREX.r2.6HG0502820), HvGAMYB1-L1 (HORVU.MOREX.r2.6HG0469220), HvGAMYB1-L2 (HORVU.MOREX.r2.7HG0544570), HvGAMYB2 (HORVU.MOREX.r2.1HG0056140), HvGAMYB (HORVU.MOREX.r2.3HG0246570), HvDcl5 (HORVU.MOREX.r2.1HG0034750), HvUPEX1 (HORVU.MOREX.r2.3HG0248470), HvUPEX2 (HORVU.MOREX.r2.1HG0053490), HvABCG23 (HORVU.MOREX.r2.1HG0046710), RBOHE (HORVU.MOREX.r2.6HG0477800), HvMAP65-3 (HORVU.MOREX.r2.6HG0458620).

## Supplementary Material

koaf230_Supplementary_Data

## Data Availability

The data that support the findings of this study are available in the Supplementary materials of this article.

## Supplementary data

The following materials are available in the online version of this article.

**Supplementary Figure S1.** RNA-seq FPKM value showing expression levels of additional putative barley bHLH TFs.

**Supplementary Figure S2.** DTT1 CRISPR target region and sequencing results.

**Supplementary Figure S3.** Comparative morphology of *dtt1-2*.

**Supplementary Figure S4.** Analysis of callose distribution in wild-type and dtt1-1 anthers.

**Supplementary Figure S5.** DTT1 and HvDYT1 subcellular localization.

**Supplementary Figure S6.** DTT1–DYT1 split YFP interactions as a positive control to determine the protein expression level under different promoters and signal of DTT1 homodimer.

**Supplementary Figure S7.** PCA **A)** and correlation matrix analysis **B)** of transcriptomic analysis data between the wild type and *dtt1-1* and *Hvtdf1-2* mutants.

**Supplementary Figure S8.** Heatmap of *K*-means cluster conducted with standard deviation, top-ranked 12,000 genes across all samples and GO term enrichment results from each cluster.

**Supplementary Figure S9.** Heatmap of gene expression pattern from PGSEA analysis results between the wild type and *dtt1-1* and *Hvtdf1-2* mutants.

**Supplementary Figure S10.** Go term analysis of biological process results of downregulated genes in *dtt1-1* mutants at stages 6, 7, and 8a1.

**Supplementary Figure S11.** Expression level of key genes in the wild type and *dtt1-1* and *Hvtdf1-2* mutants from RNA-seq data.

**Supplementary Figure S12.** Identified key factors downregulated during anther transition from stage 6 to stage 7.

**Supplementary Figure S13.** Phylogenetic analysis of reported GAMYBs from rice, *Arabidopsis*, and identified barley genes.

**Supplementary Figure S14.** Negatively enriched motifs from 3×HA negative control.

**Supplementary Figure S15.** The enriched reads with dual-E-box motifs from the DTT1–HvDYT1 complex.

**Supplementary Figure S16.** Dual-luciferase assay of the DTT1–DYT1 complex showing activation gene expression.

**Supplementary Figure S17.** Testing promoters from putative targets via dual-luciferase assay.

**Supplementary File 1.** FASTA file of the phylogenetic tree alignment.

**Supplementary File 2.** Newick file of the phylogenetic tree alignment.

**Supplementary Table S1.** Barley DEGS with *P* = 0.05 and log2FC − 4.

**Supplementary Table S2.** The annotated unique genes shared between *dtt1-1* downregulated and *Hvtdf1-2* downregulated genes.

**Supplementary Table S3.** Primers and constructs used in this study.

**Supplementary Table S4.** Statistical analysis results summary of Supplementary Fig. S16.

## Dive Curated Terms

The following phenotypic, genotypic, and functional terms are of significance to the work described in this paper:

TIP2 Gramene: AT3G26520
TIP2 Araport: AT3G26520

PIF4 Gramene: AT2G43010
PIF4 Araport: AT2G43010

SERK2 Gramene: AT1G34210
SERK2 Araport: AT1G34210

rpk2 Gramene: AT3G02130
rpk2 Araport: AT3G02130

MYC2 Gramene: AT1G32640
MYC2 Araport: AT1G32640

TDF1 Gramene: AT3G28470
TDF1 Araport: AT3G28470

DCL5 Gramene: Os10g0485600
DCL5 Araport: Os10g0485600

MYB80 Gramene: AT5G56110
MYB80 Araport: AT5G56110

bHLH089 Gramene: AT1G06170
bHLH089 Araport: AT1G06170
