# Supplementary material for: The basic helix–loop–helix (bHLH) transcription factor DTT1 is part of a paired key that unlocks the tapetum transition in barley anther development
Source: Plant Cell. 2025 Sep 30;37(10):koaf230. doi: 10.1093/plcell/koaf230 (PMC12510322; doi:10.1093/plcell/koaf230)

Supplemental Data

Fig. S1. RNAseq FPKM value showing expression levels of additional putative barley bHLH transcription factors.

(A) HvEAT1-L1, (B) HvEAT1-L2, (C) DTT1. Error bars indicate SD from two biological replicates. Stage: St. This data supports Fig. 1.

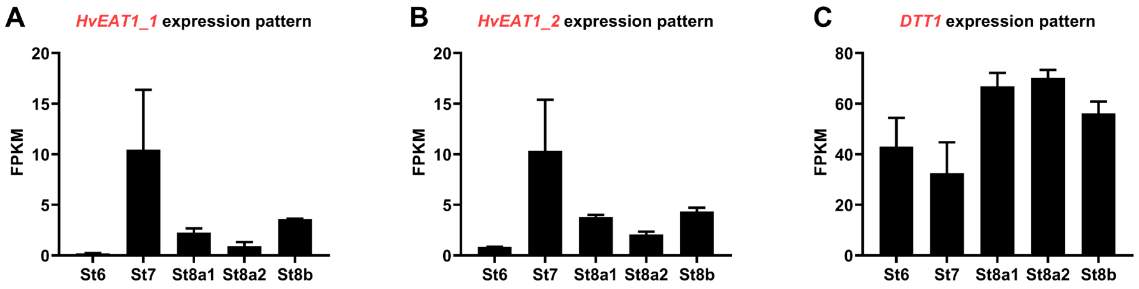

Fig. S2. DTT1 CRISPR target region and sequencing results

(A). Structure of the *DTT1* gene from exon 1 to exon 2, nuclei acid sequences underneath the gene structure are the sgRNA target regions with turquoise highlight and red highlight for protospacer-adjacent motif (PAM).

(B). The *dtt1-1*: 229 bp deletion from 190 bp to 418 bp on gDNA in Red.

(C). PCR results showing *dtt1-1* homozygous mutants (lanes 1-12) and wild type (WT).

(D). The *dtt2*-2: 2bp deletion at 190 bp in Red and 1bp insertion at 418bp in Blue. This data supports Fig. 2.

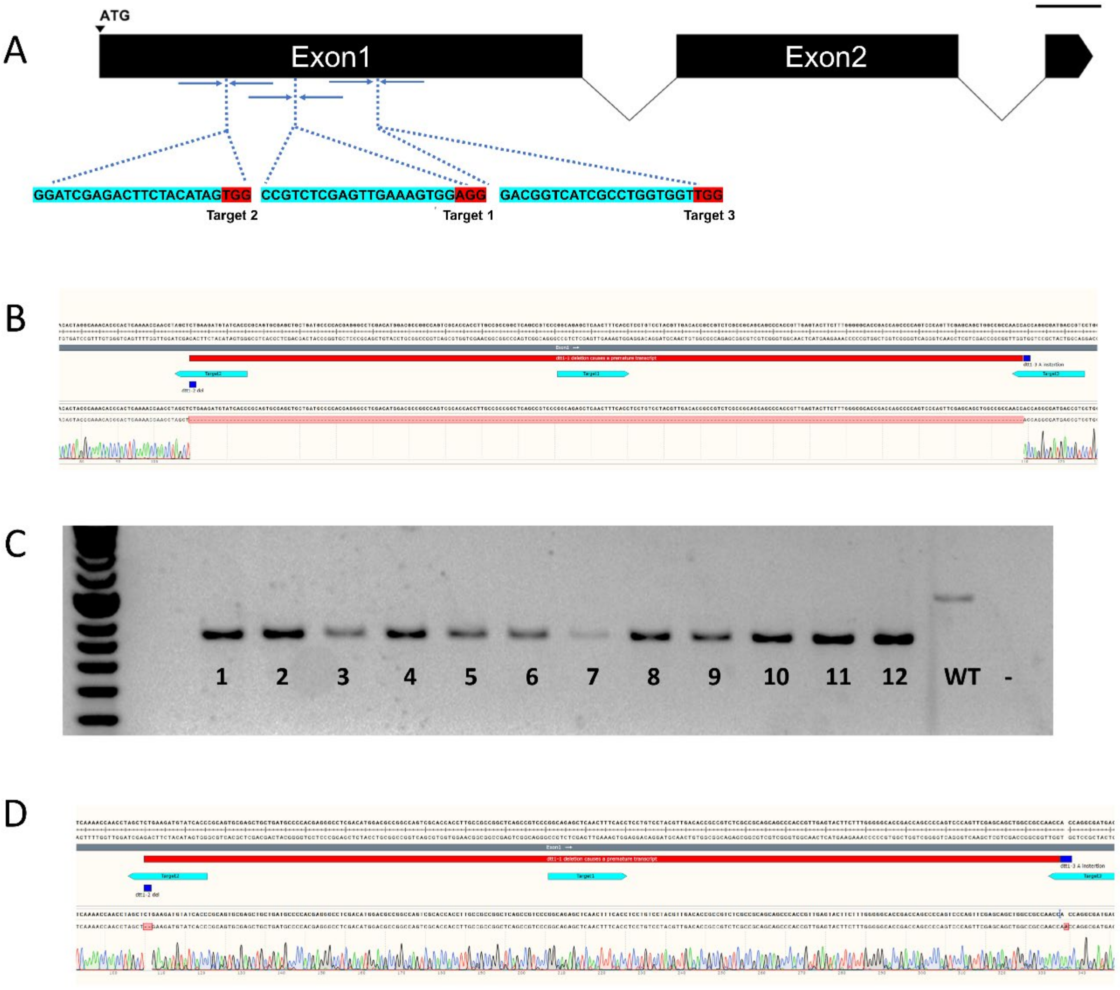

Fig. S3. Comparative morphology of *dtt1-2*

(A-B) Whole plant morphology, (A) Wild type, (B) *dtt1-2,* Bar=50cm. (C-D) Morphology of floret with lemma and palea removed, (C) Wild type, (D) *dtt1-2,* Bar=1mm. (E-F). KI-I2 pollen viability, (E) Wild type, (F) *dtt1-2,* Bar=2mm. (G). Mature spikes, Wild type (left), *dtt1-1* (middle) and *dtt1-2* (right). Bar=5 cm. This data supports Fig. 2.

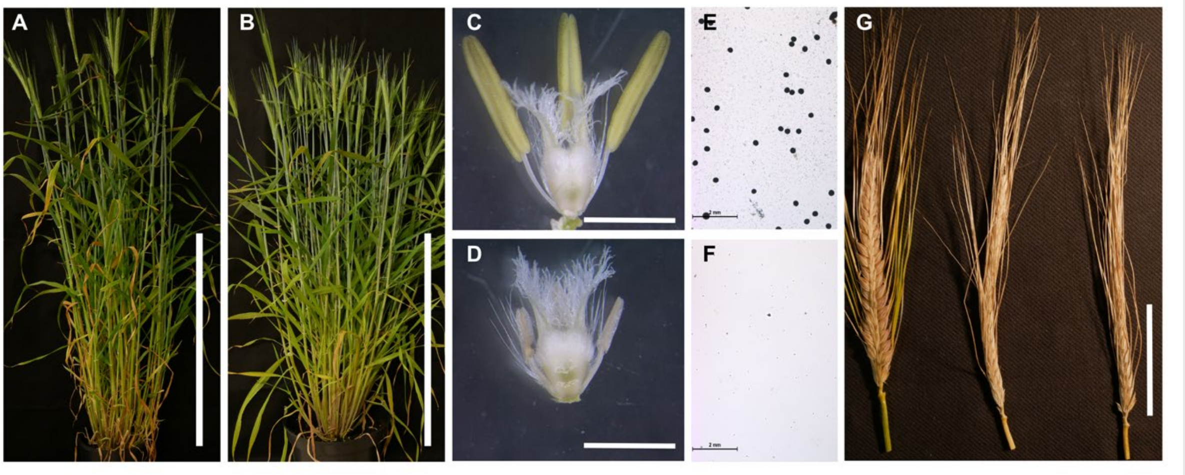

Fig. S4. Analysis of callose distribution in wild type and *dtt1-1* anthers.

(A) and (B). Wild type anther stained with aniline blue at stage 8b and stage 9, respectively.

(C) and (D). *dtt1-1* anther stained with aniline blue at stage 8b and stage 9, respectively. The cyan colour indicates the signal from the stained callose; the purple colour shows the autofluorescence of barley anthers. The wild type tetrads and newly released single microspores are surrounded by callose, however the mutant line shows irregular callose accumulation in the middle of the anther. Bars = 30 μm. This data supports Fig. 3.

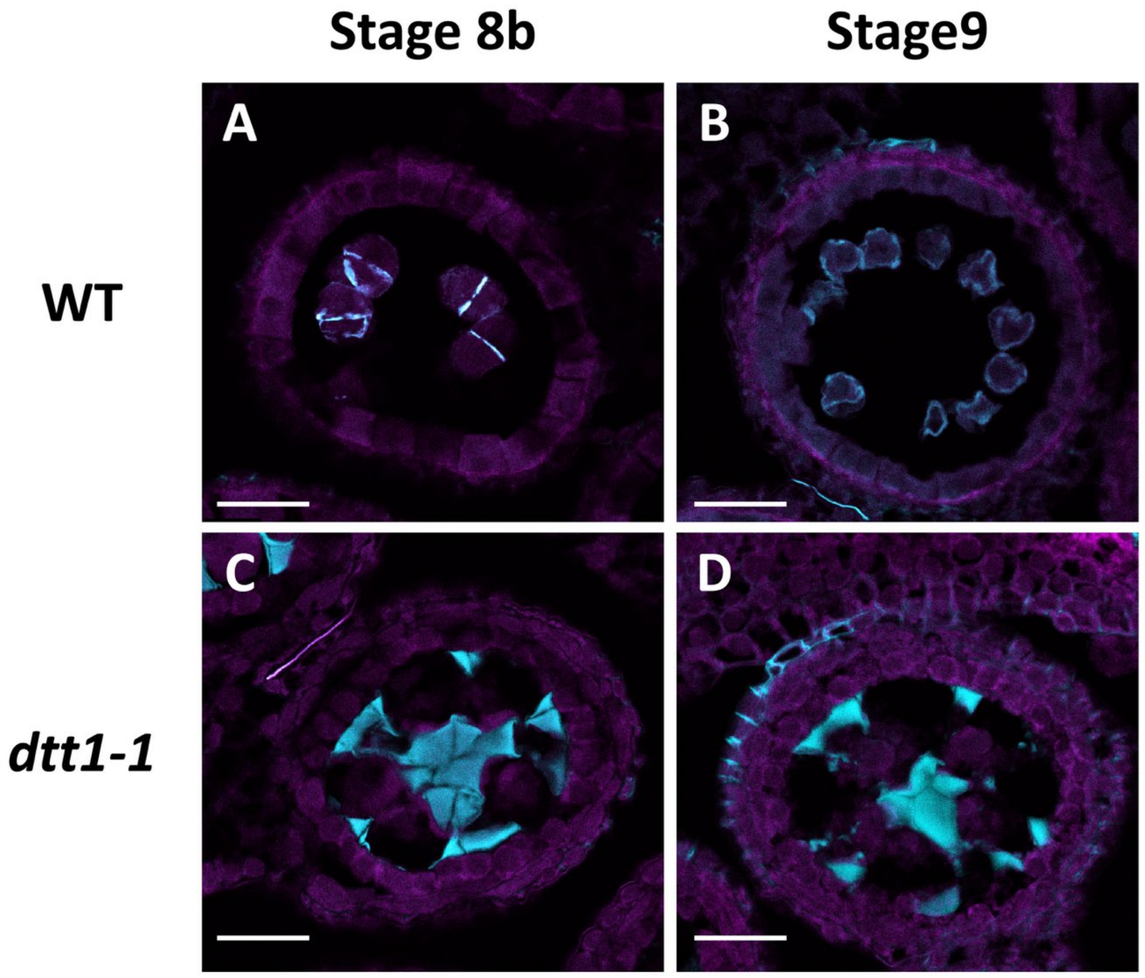

Fig. S5. DTT1 and HvDYT1 subcellular localization

(A) to (C). Subcellular protein localization of HvDTT1-GFP protein in *Nicotiana benthamiana* leaves. (A) DAPI stained nuclei, (B) HvDTT1-GFP signal, and (C) merged signal from (A) and (B). Bars= 25 µm. (D) to (F). Subcellular protein localization of HvDYT1-GFP protein in *Nicotiana benthamiana* leaves. (D) DAPI stained nuclei, (E) HvDYT1-GFP signal, and (F) merged signal from (D) and (E). Bars= 25 µm. This data supports Fig. 5.

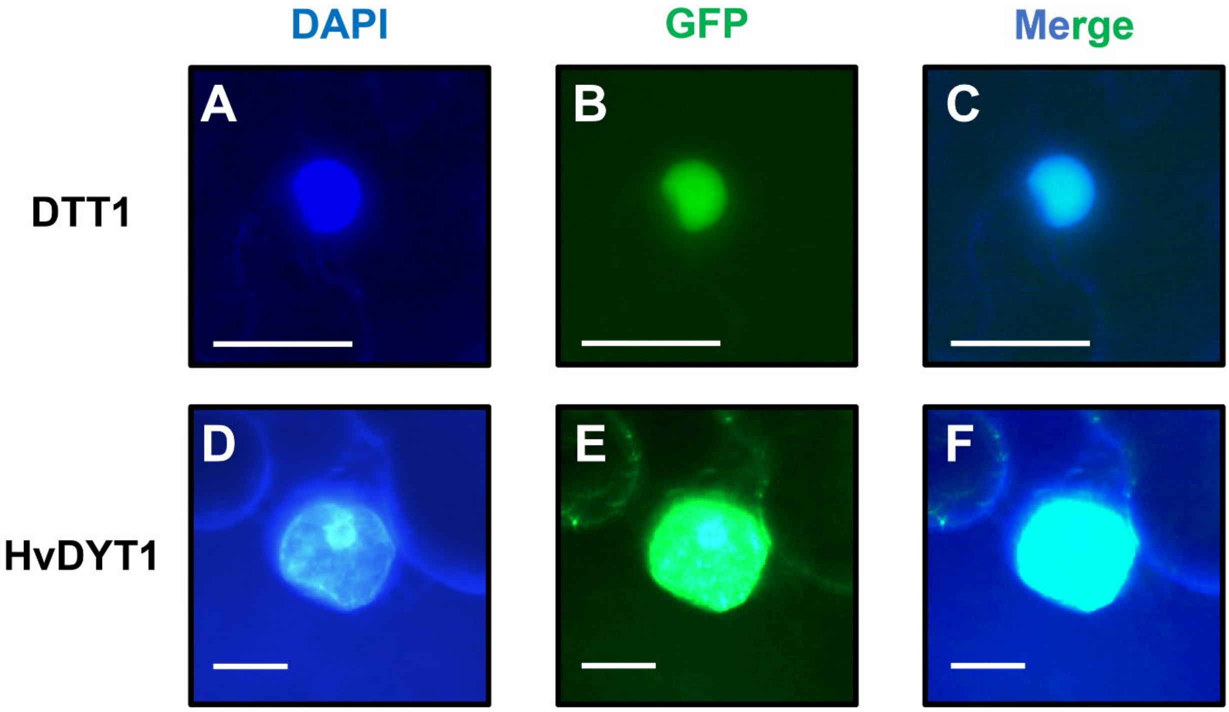

Fig. S6. DTT1-DYT1 split YFP interactions as a positive control to determine the protein expression level under different promoters and signal of DTT1 homodimer.

(A). The signal of the interacted *AtUBQ10*promoter-driven DTT1 and *AtUBQ10*promoter-driven DYT1. Bars=100 μm (B). The signal of the interacted *AtUBQ10*promoter-driven DTT1 and 2xCaMV35s promoter-driven DYT1. Bars=100 μm (C). The signal of the interacted 2xCaMV35s-driven DTT1 and 2xCaMV35s promoter-driven DYT1. Bars=100μm. (D). The homodimer detected with increased expression level of DTT1 with 2xCaMV35s promoter. Bars=20 μm. (E). The increased expression of DTT1-△1 showed interaction with DYT1. Bars=20 μm. (F). The increased expression of DTT1-△2 showed interaction with DYT1. Bars=20 μm. This data supports Fig. 5.

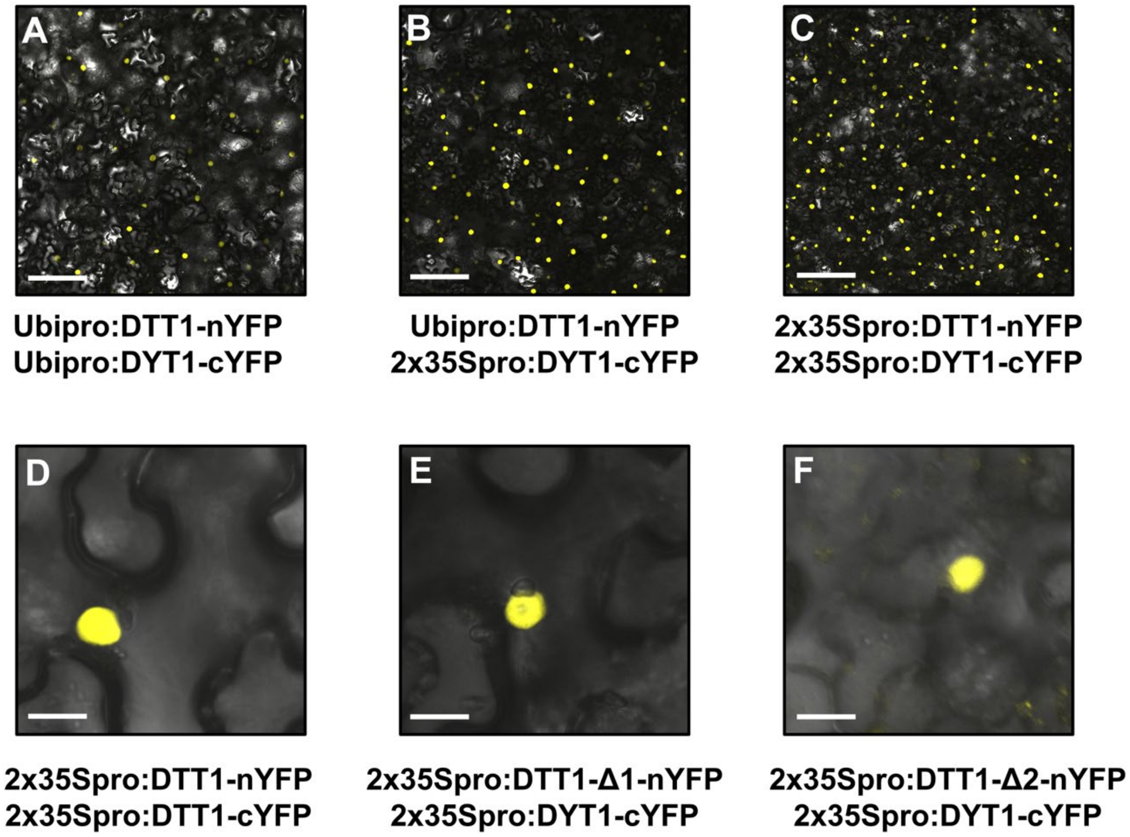

Fig. S7. Principal component analysis (A) and correlation matrix analysis (B) of transcriptomic analysis data between the wild type and *dtt1-1* and *Hvtdf1-2* mutants. This data supports Fig. 7.

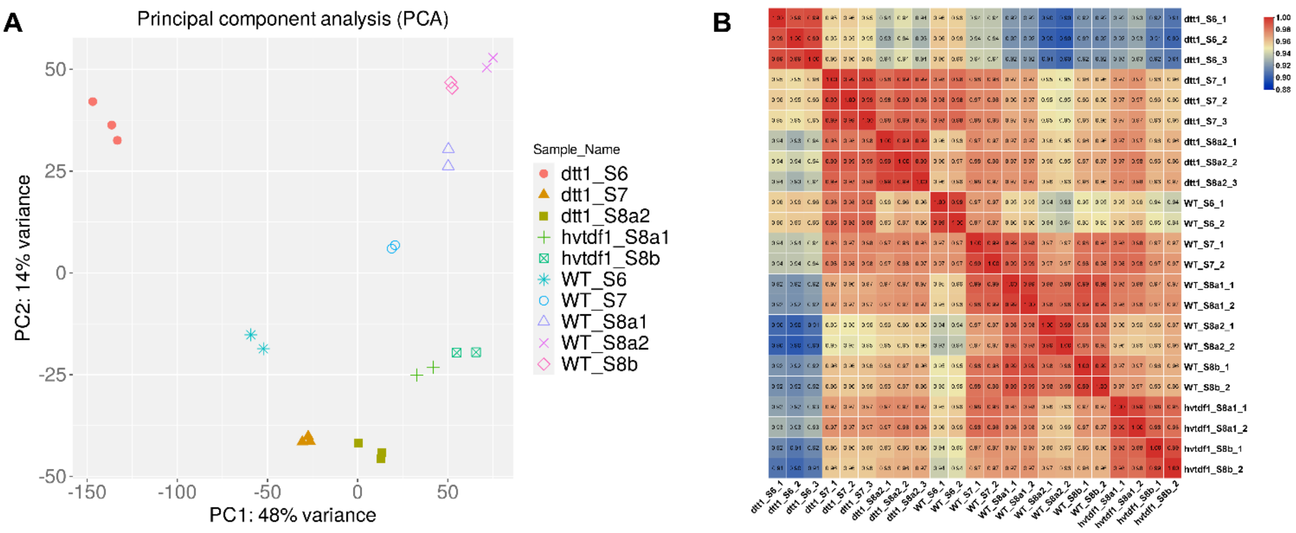

Fig. S8. Heatmap of K-means cluster conducted with standard deviation, top ranked 12,000 genes across all samples and GO Term enrichment results from each cluster.

(A) K-means clustering heatmap from all samples, (B) GO Term enrichment of Biological Process of cluster 1 genes, (C) GO Term enrichment of Biological Process of cluster 2 genes, (D) GO Term enrichment of Biological Process of cluster 3 genes, (D) GO Term enrichment of Biological Process of cluster 4 genes. This data supports Fig. 7.

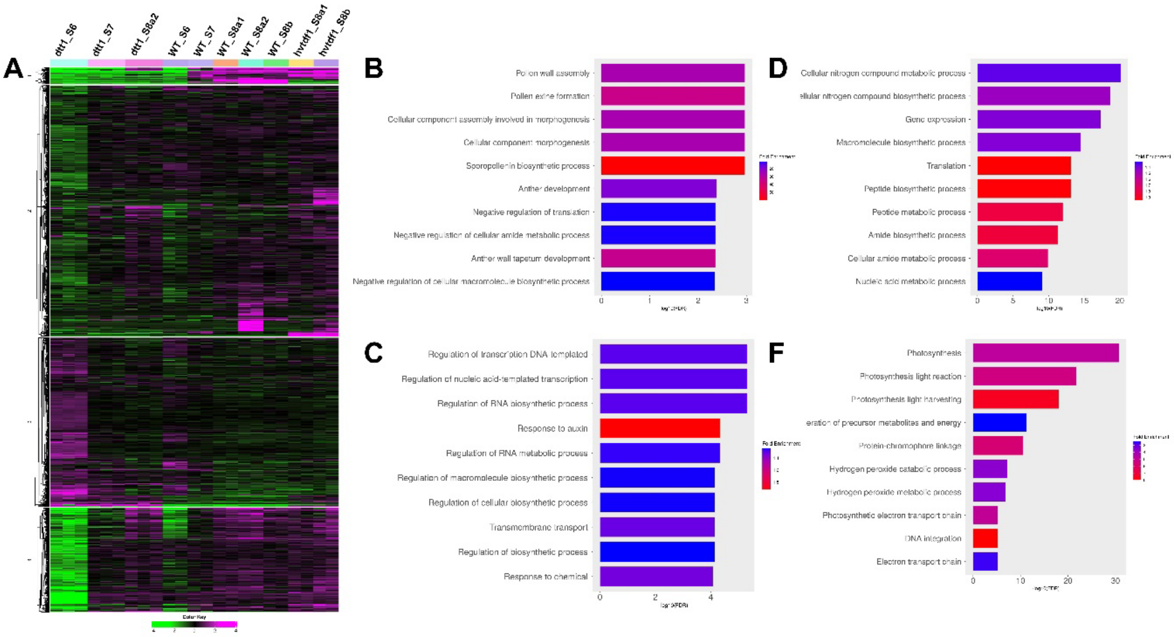

Fig. S9. Heatmap of gene expression pattern from PGSEA analysis results between the wild type and *dtt1-1* and *Hvtdf1-2* mutants. This data supports Fig. 7.

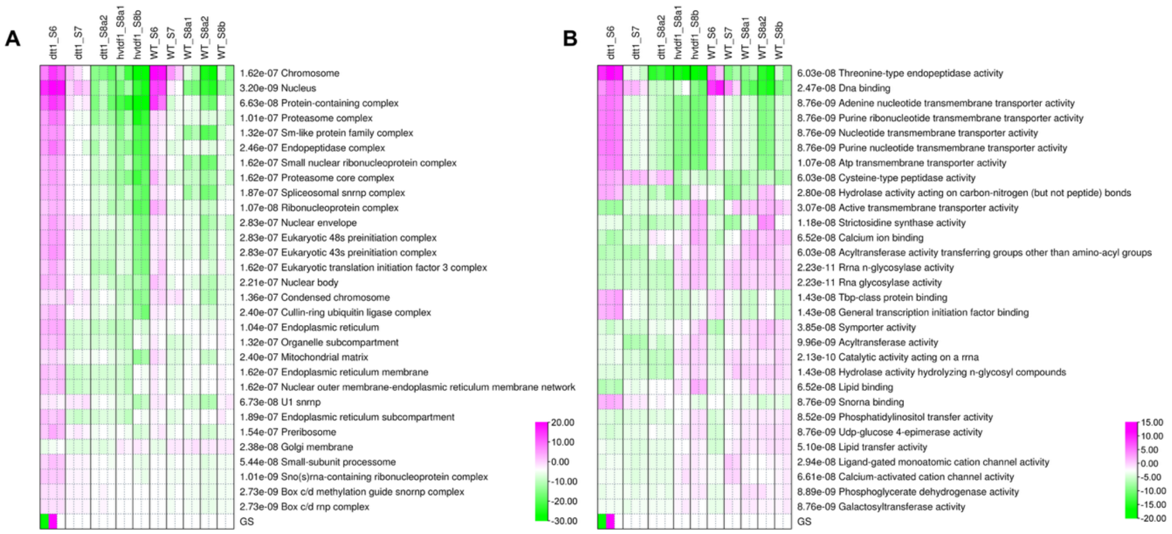

Fig. S10. Go Term analysis of biological process results of down-regulated genes in *dtt1-1* mutants at stages 6, 7, and 8a1.

(A) Stage 6, (B) Stage 7, (C) Stage 8a1, (D) Venn diagram results of down-regulated genes in *dtt1-1* mutants at stage 6, stage 7, and stage 8a1. This data supports Fig. 7.

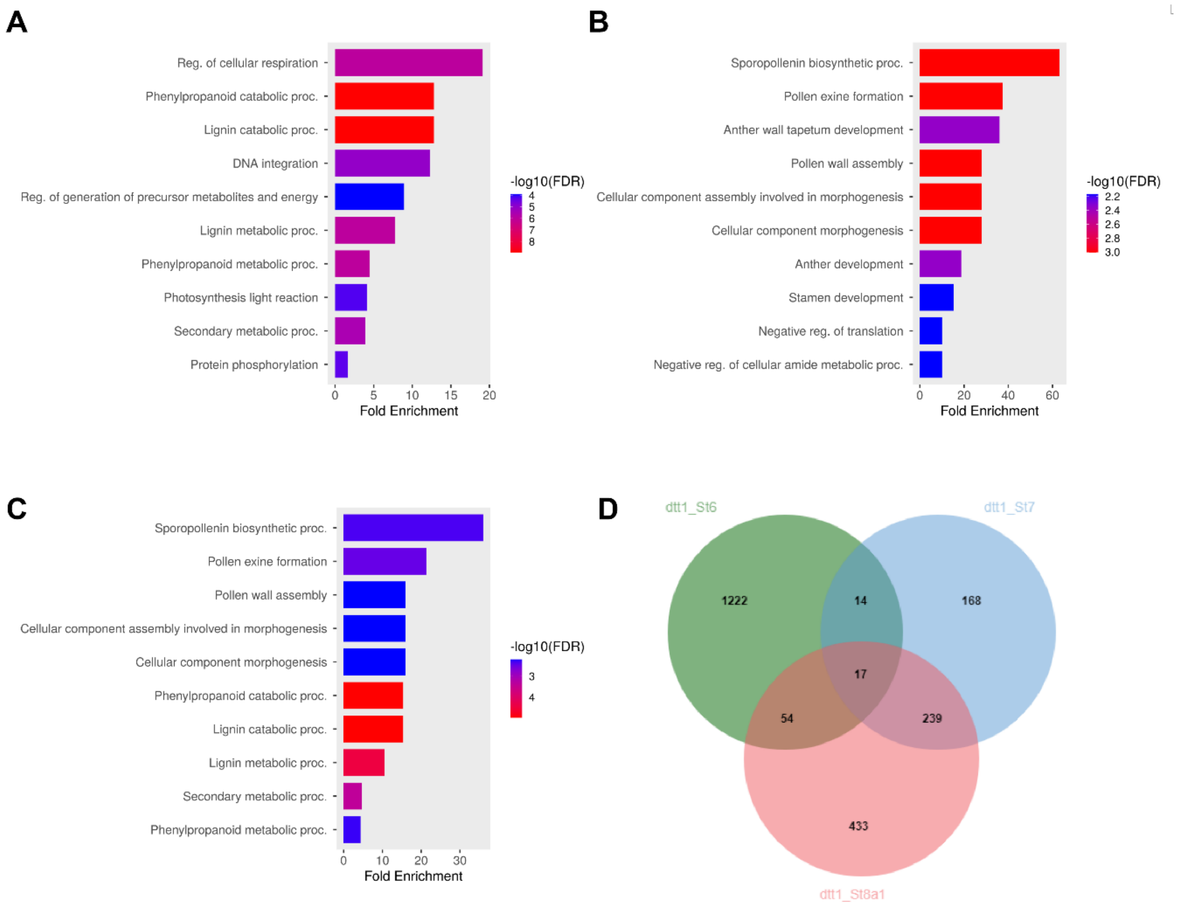

Fig. S11. Expression level of key genes in the wild type and *dtt1-1* and *Hvtdf1-2* mutants from RNA-seq data.

(A) *HvDYT1*, (B) *DTT1*, (C) *HvTDF1*, (D) *HvAMS*, (E) *MYB80*, (F) *HvEAT1_L1*, (G) *HvEAT1_L2*, (H) *HvGAMYB*, (I) *HvGAMYB2*, (J) *HvGAMYBL1*, (K) *HvGAMYBL2*, (L) *HvGASA8*, (M) *HvDcL5*, (N) *HvABCG23*, (O) *RBOHE*, (P) *HvMAP65-3*. Error bars represent SD from 3 biological replicates. This data supports Fig. 8.

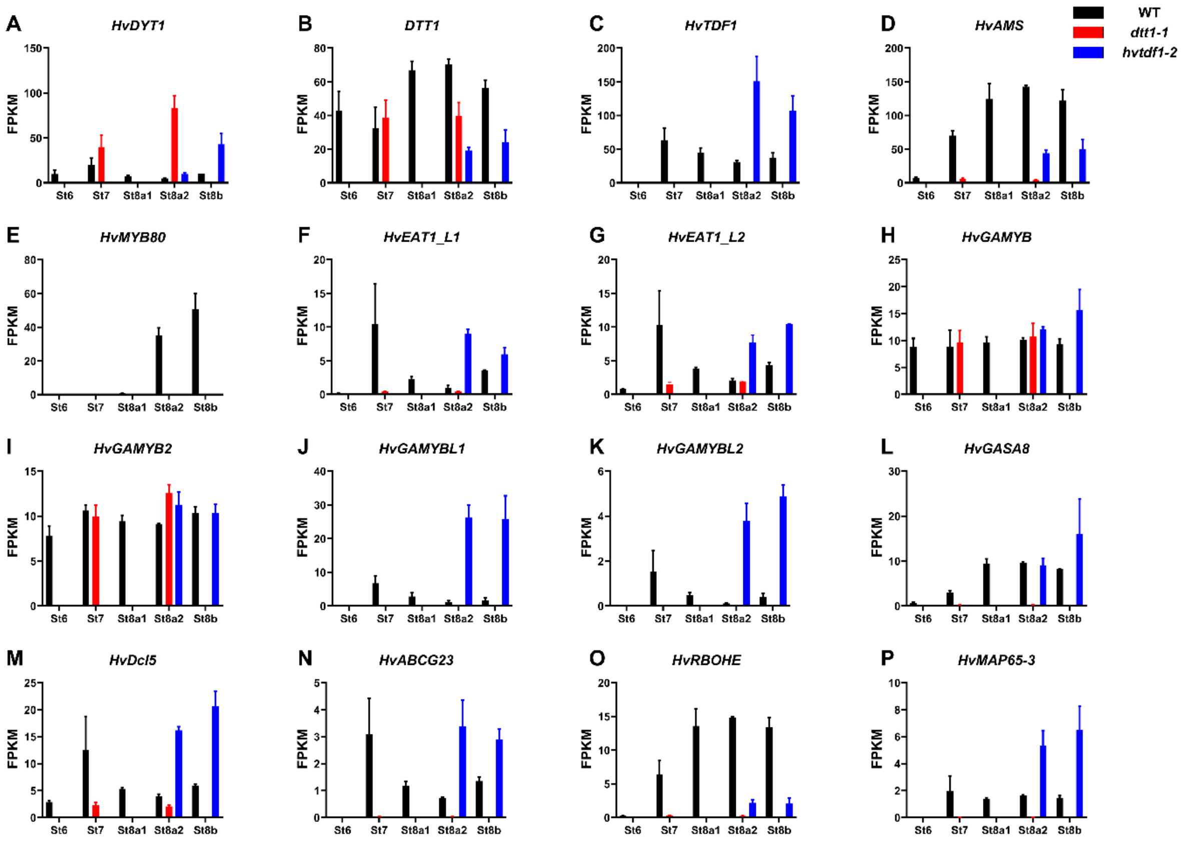

Fig. S12. Identified key factors down-regulated during anther transition from stage 6 to stage 7.

(A) Venn diagram analysis of down-regulated between *dtt1-1* and *Hvtdf1-2*, (B) GO Term analysis of biological processes of *dtt1-1* unique down-regulated genes. This data supports Fig. 7.

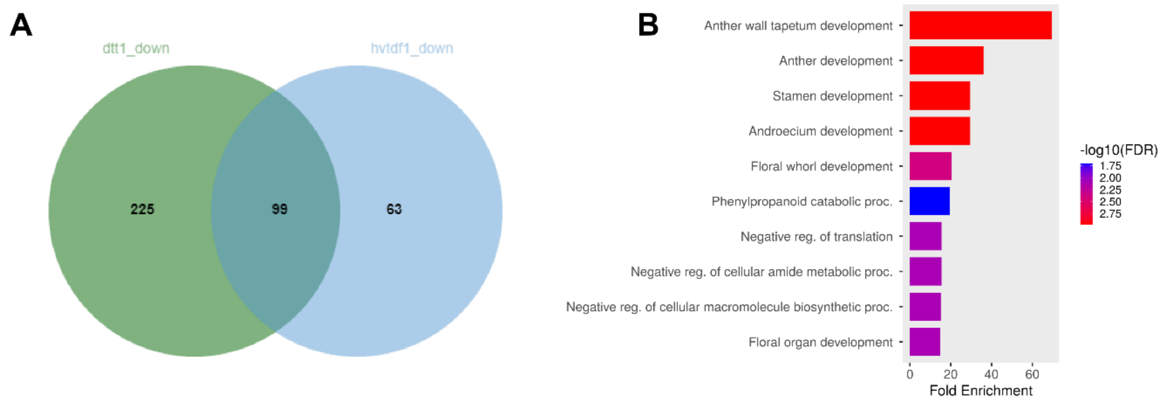

Fig. S13. Phylogenetic analysis of reported GAMYBs from rice, Arabidopsis and identified barley genes. The values represent the Bootstrap value. This data supports Fig. 8.

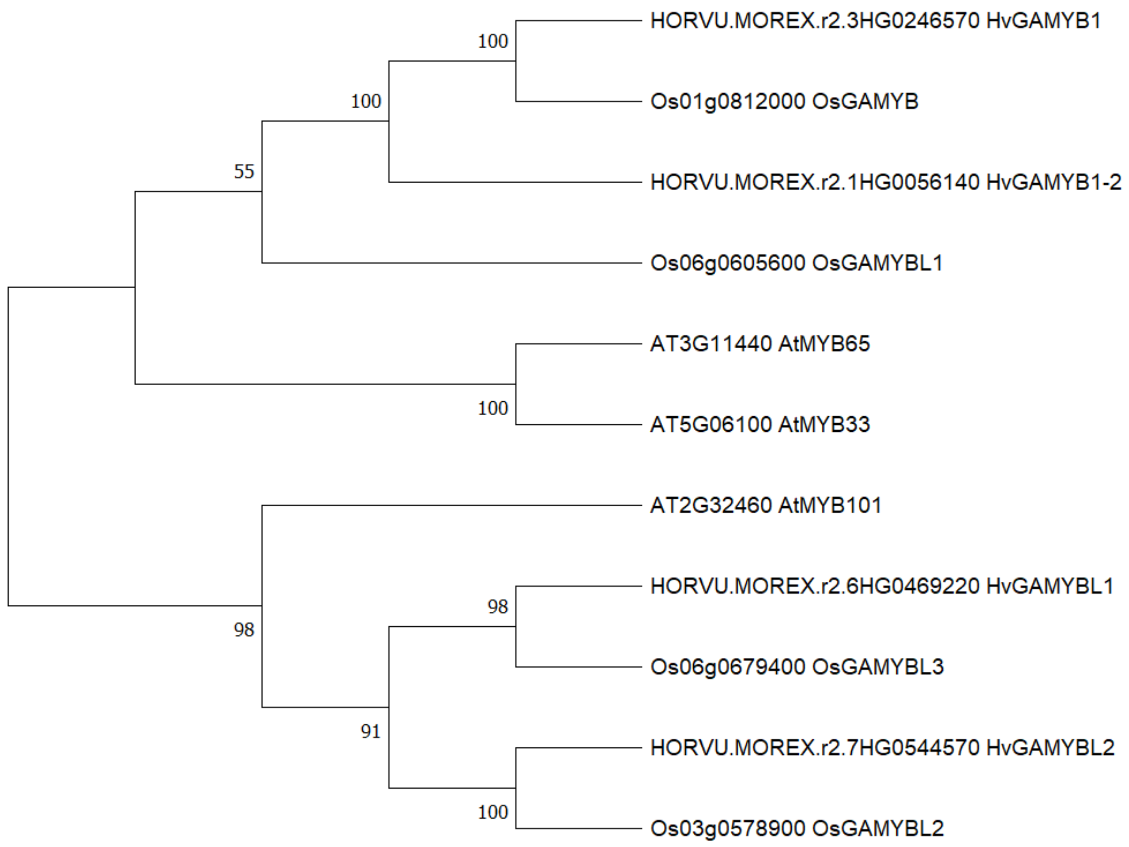

Fig. S14. Negatively enriched motifs from 3xHA negative control. This data supports Fig. 8.

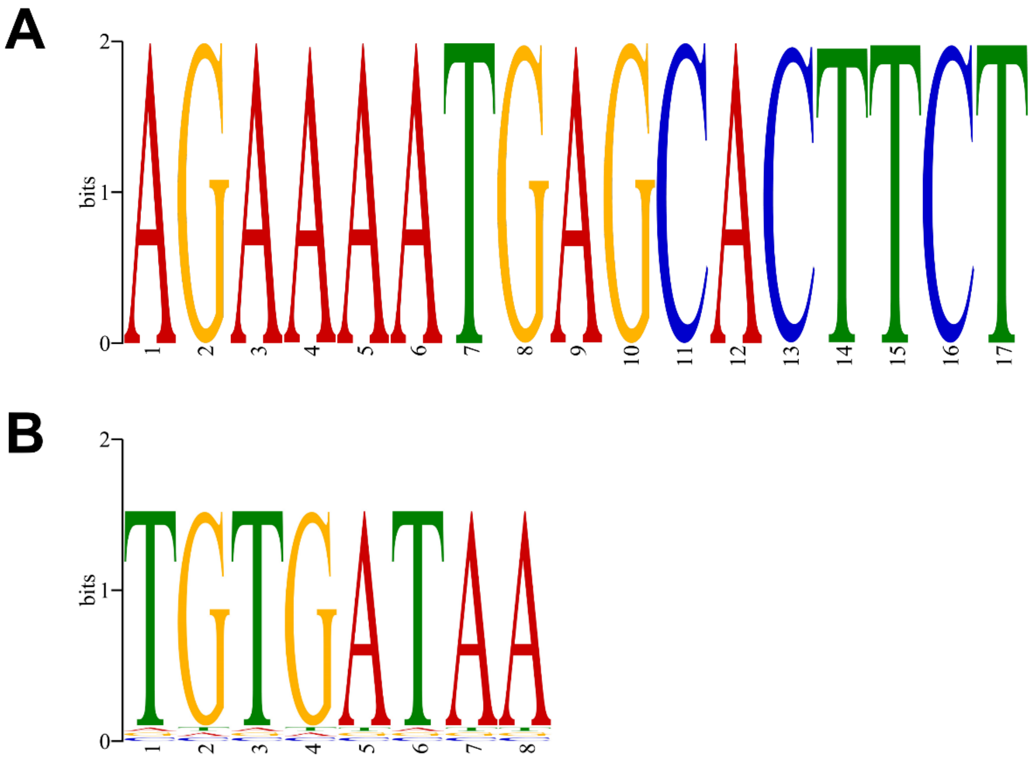

Fig. S15. The enriched reads with Dual E-box motifs from DTT1-HvDYT1 complex.

(A). The reads with canonical E-box motif in same orientation

(B). The reads with canonical E-box motif in inverse orientations

(C). The reads with canonical E-box and non-canonical E-box

(D). The reads with canonical E-box and non-canonical E-box

Green color highlighted the CACGTG canonical E-box and Pink color is in inverse orientation; Turquoise indicating the non-canonical E-box (HRE motif); yellow highlights the repeated non-canonical E-box. This data supports Fig. 8.

A

TCACGTGCAACACGTGTCCCACTAT
GTCACGTGAAACACGTGCACCTCGC
GTCACGTGAAACACGTGATTGCGTA
GGCACGTGAAACACGTGACTCGACG
GGCAACACGTGTAACACGTGACGAA
GCGCACGTGAACCACGTGATAGCTT
GCAGTGAGACACGTGCAACACGTGT
GAGCACTATTCACGTGCAACACGTG
GACACGTGAACCACGTGATGTACTC
CCCATCACGTGCAACACGTGACTCC

B

GCTTACGTGCACTTTGACACGTGCC
GCTTACGTGCACTATGACACTTGAC
GCAGTCACGTGCTTTACGTGCACAG
CTGTGCACGCGAATCACGTGATCGT
CTCACGTGGCTTCGCTTACGTGCAC
CTCACGTGGCTCTGCTTACGTGCAC
CTCACGTGCGTTCGCTCACGTGCAC
CTCACGTGCCTTCTCTTACGTGCAC
CTCACGTGCCTTCGTTTACGTGCAC
CGTGCACTCATGCACAACACGTGAT

C

TCTTACGTGCTCTTTGACACGTGCC
GCTTACGTGTTCTTTGACACGTGCC
TTCACGTGCCTTCGCTTACGTGCAC
TCACGTGTTATACGTGTGTCGCCTC
GTTTACGTGCTCTTTGACACGTGCC
GTGTCACGTGTTGTACGTGCTGTCG
GTGTAACACACGTGCCTTACGTGAC
CTTACGTGCCTTCGCTTACGTGCAC
CACGTGCACGAGCAGGCTTACGTGC
AAGTGCATGTAAGCGAAGGCACGTG

D

GGATCACGTGAACTTCGTGTTTAGG
AATCACGTGTTGTTCGTGCTTCGTG
AAGTATAGGTGTCACGTGTTTCGTG
AAGAACAGTCACGTGTTGTTCGTGC
AACATCACGTGTTTCGTGTGACTAC
AACACGTGTTTCGTGTGCTTCATC
AGAGCACACGTGTTATTCGTGACAA
AAGTCACGTGCTTCGTGTGAACCGG
AAAGGGATACGTCACGTGTTTCGTG

Fig. S16. Dual luciferase assay of the DTT1-DYT1 complex showing activation gene expression.

The relative reporter activity of transcription factor activation assay of DYT1 homodimer, DTT1-DYT1 heterodimer. The black asterisks indicate the statistically significant compared with HvDYT1 homodimer. (P<0.01, t-test). Error bars indicate SD of the biological replicates, n =6. This data supports Fig. 8.

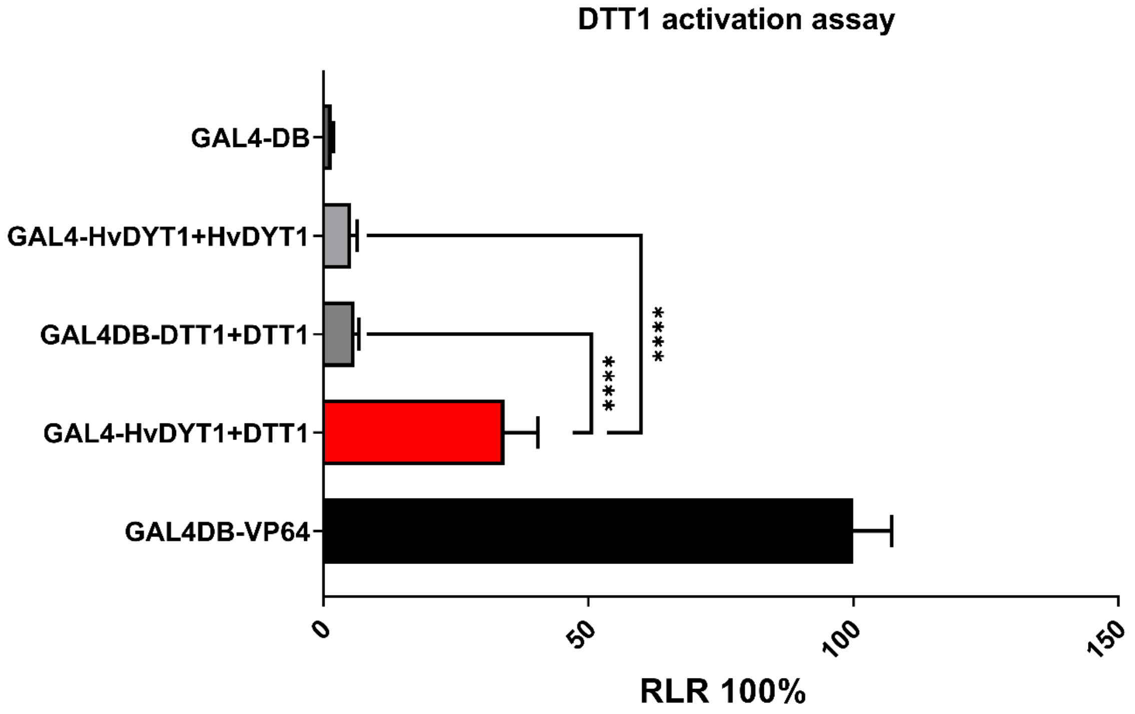

Fig. S17. Testing promoters from putative targets via dual-luciferase assay. This data supports Fig. 8.

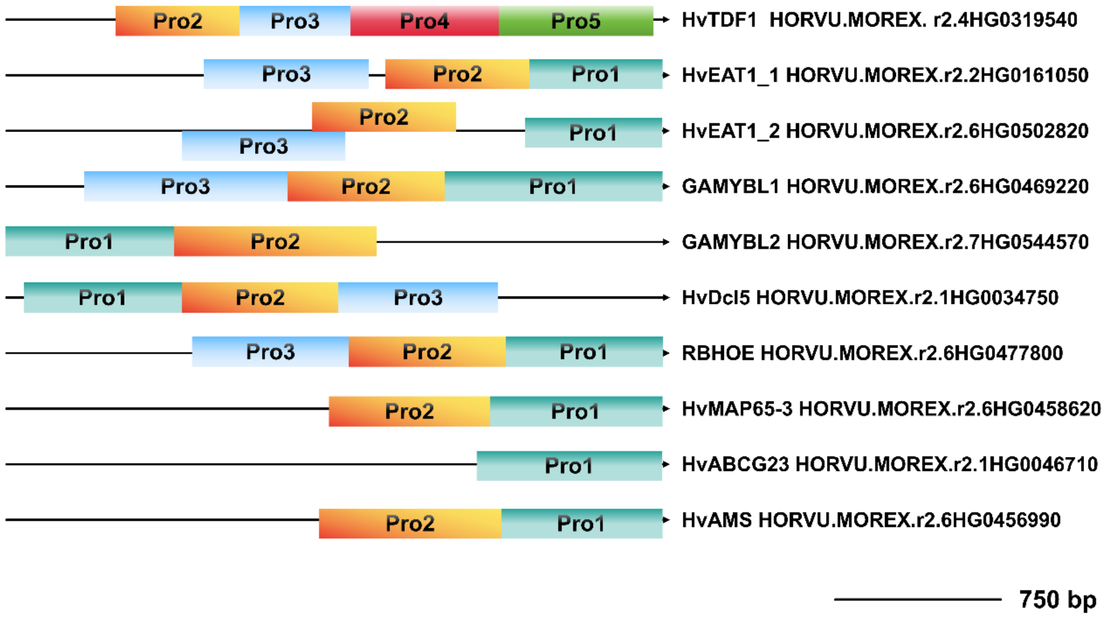

Supplement: koaf230_Supplementary_Data [file koaf230_supplementary_data.zip › Supplemental Data-Final Sept 2025_2.pdf]
